# Supplementary material for: Lentivirus-mediated gene therapy for Fabry disease
Source: Nat Commun. 2021 Feb 25;12:1178. doi: 10.1038/s41467-021-21371-5 (PMC7907075; doi:10.1038/s41467-021-21371-5)
Supplement: Supplementary file 1 — Supplementary Information [file 41467_2021_21371_MOESM1_ESM.pdf]

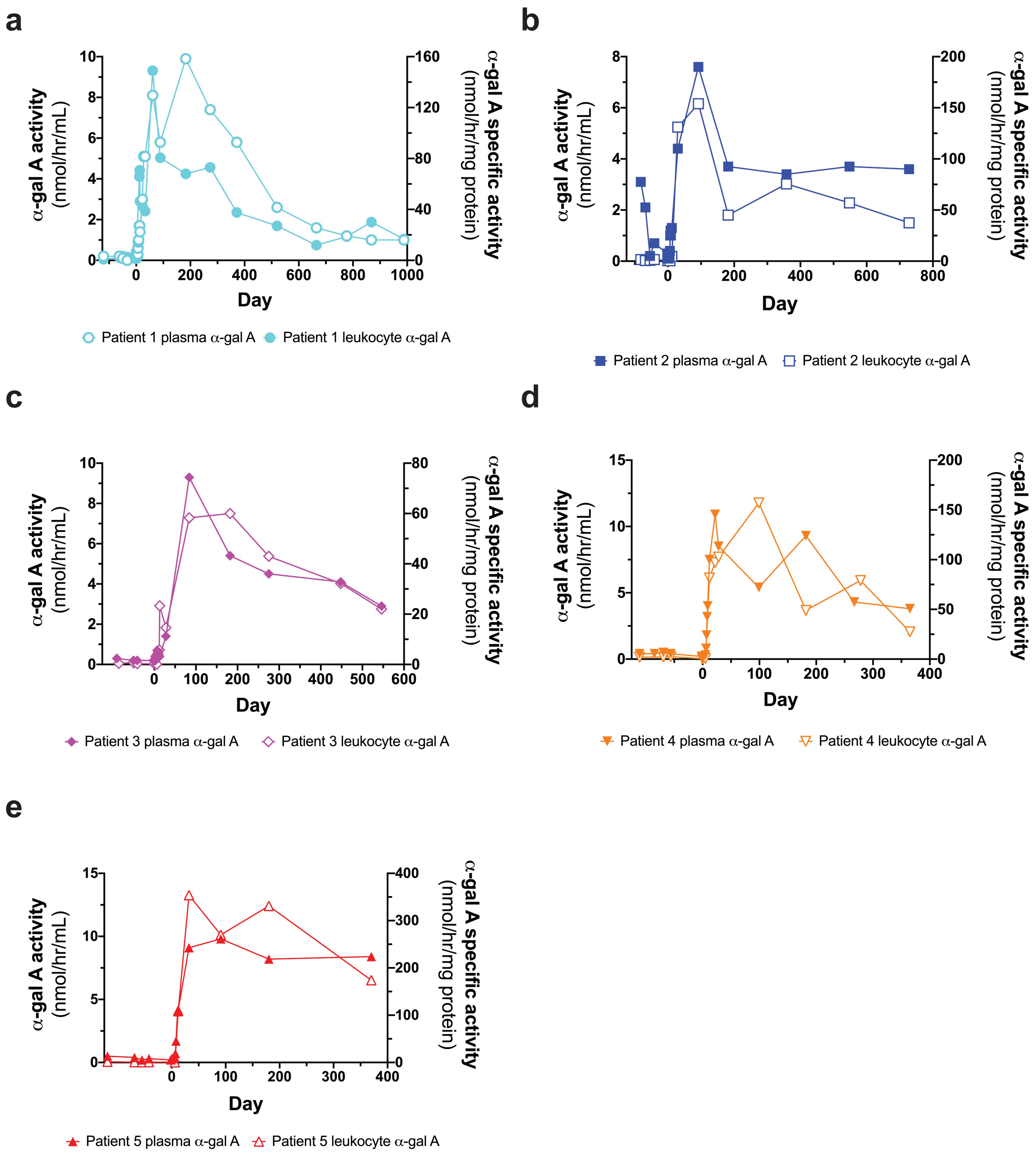

Supplementary Figure 1. Plasma α-Galactosidase A (α-gal A) Activity and Leukocyte α-gal A Specific Activity.

Plasma α-gal A activity and leukocyte α-gal A specific activity are shown for each patient: a) Patient 1, b) Patient 2, c) Patient 3, d) Patient 4 and e) Patient 5. Plasma α-gal A activity is similar to leukocyte α-gal A specific activity.

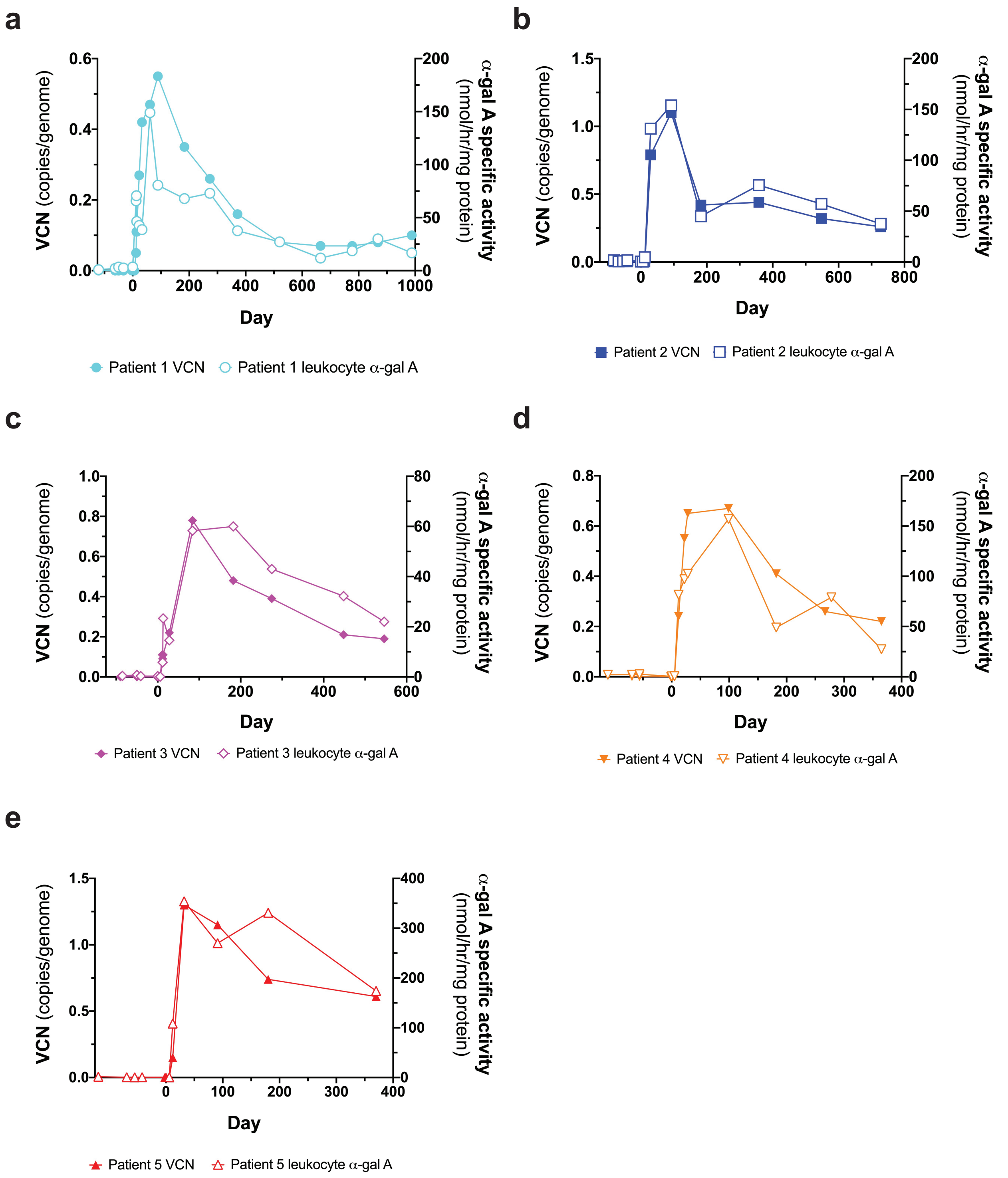

Supplementary Figure 2. Vector Copy Number (VCN) versus Leukocyte α-Galactosidase (α-gal A) Specific Activity.

VCN and leukocyte α-gal A specific activity are illustrated for each patient: a) Patient 1,b) Patient 2, c) Patient 3, d) Patient 4 and e) Patient 5. VCN mirrors leukocyte α-gal A specific activity for all patients.

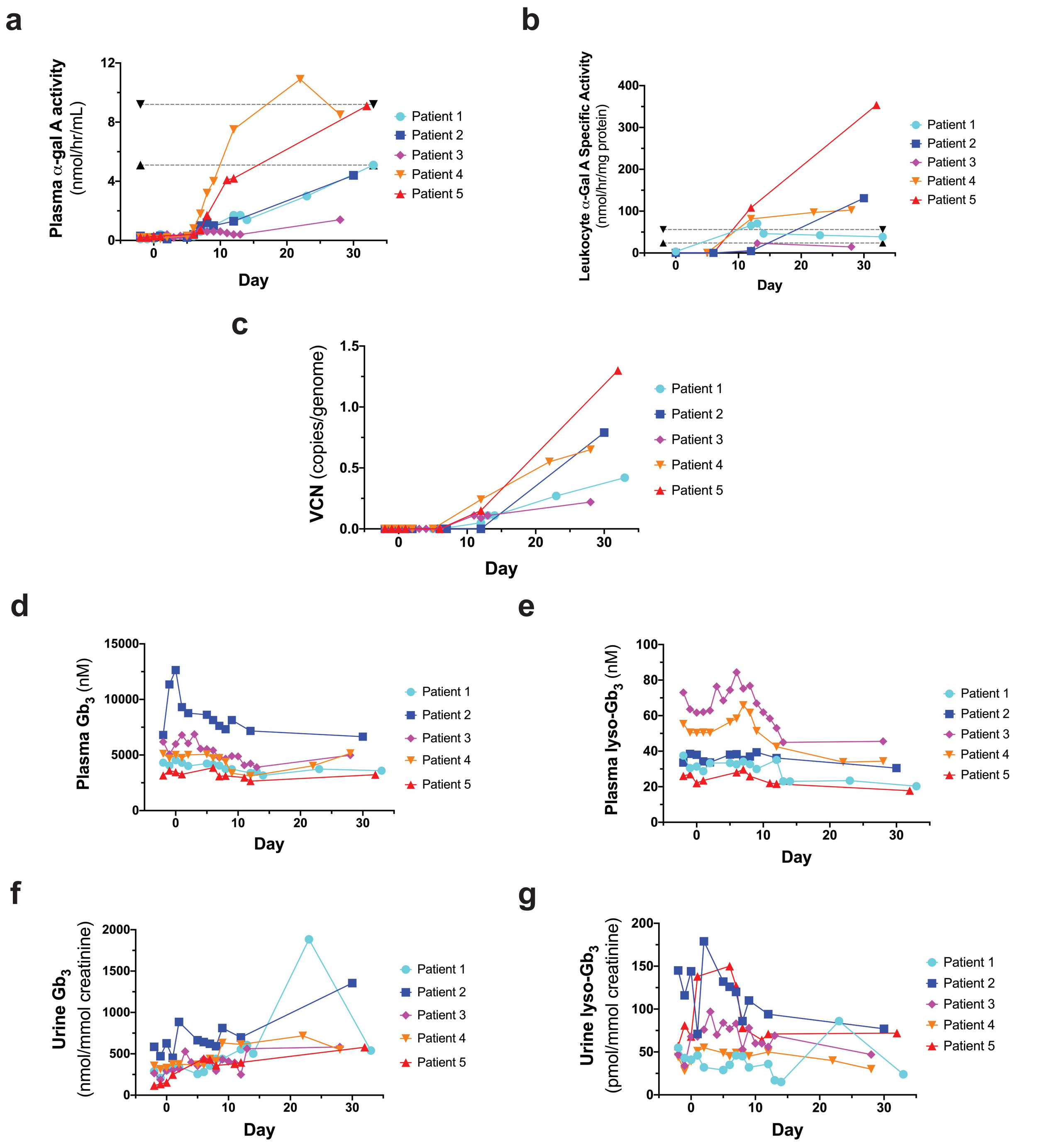

Supplementary Figure 3. Treatment Phase Data.
Plasma α-galactosidase A (α-gal A) activity (a), leukocyte α-gal A specific activity (b), vector copy number (VCN) (c), plasma Globotriaosylceramide (Gb3) (d), plasma lyso-Gb3 (e), urine Gb3 (f) and urine lyso-Gb3 (g) data are illustrated from Day -2 to Day 30. The reference ranges for plasma α-gal A (A) and leukocyte α-gal A activity (B) are indicated by dotted lines.

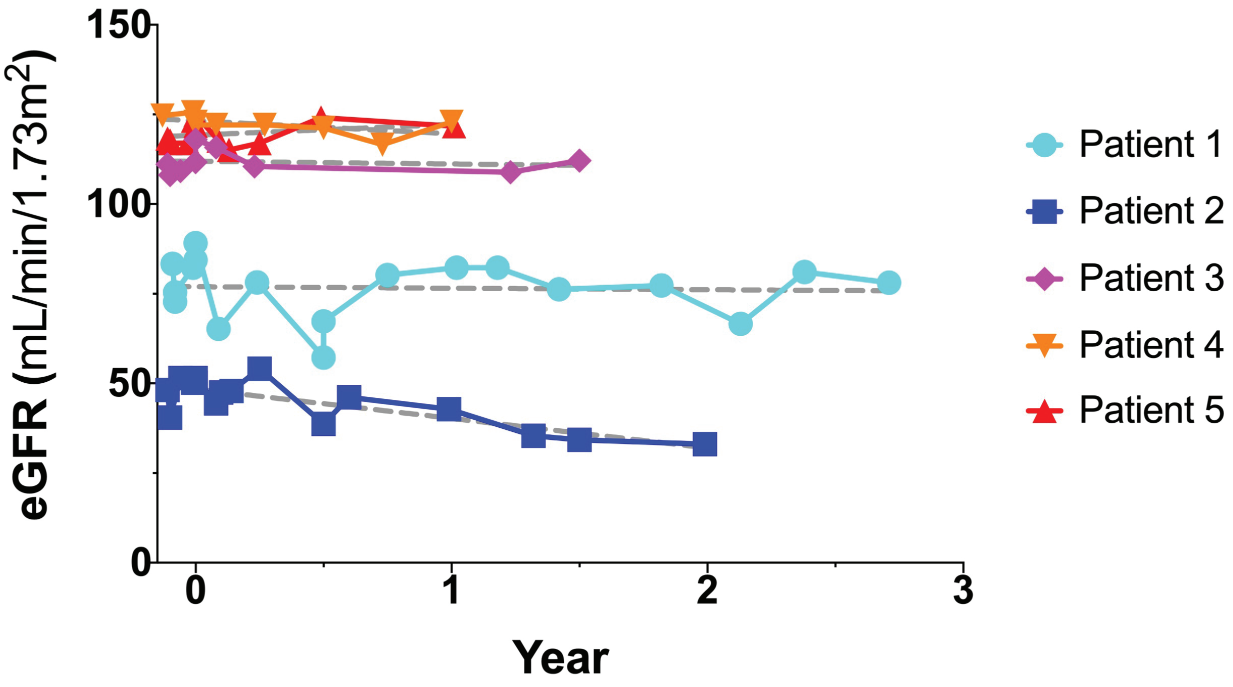

eGFR/year

**Patient 1**	**-0.43**
**Patient 2**	**-8.22**
**Patient 3**	**-0.75**
**Patient 4**	**-3.61**
**Patient 5**	**3.01**

Supplementary Figure 4. estimated Glomerular Filtration Rate (eGFR) Slopes
eGFR data corresponding to all data points except from Day 0-28 are illustrated. Linear regression was performed. The eGFR was divided by the total study period to derive eGFR/year.

Supplementary Table 1. Additional Baseline Patient Demographics.

**Parameter**	**Patient 1**	**Patient 2**	**Patient 3**	**Patient 4**	**Patient 5**
**Age started ET (years)**	36/40	33/39	36	26	29
**Source of ET**	FABRAZYME/ REPLAGAL	REPLAGAL/ FABRAZYME	REPLAGAL	REPLAGAL	FABRAZYME
Other Symptoms at Baseline					
**Allergy**	Raw onions	Sulfa drugs			
**Anemia**				X	
**Asthma**					X
**Cellulitis**					X
**Chiari malformation**			X		
**Depression**					X
**Dizziness**			X		
**Dyspnea**					X
**Eczema**					X
**Edema (limbs)**		X			X
**Gynecomastia**					X
**Hypertriglyceridemia**				X	
**Hypophosphatemia**					X
**Insomnia**	X				
**Non-cardiac chest pain**					X
**Sinus bradycardia**			X	X	
**Sleep apnea**					X
**Urgency**	X				
**CD34+ cells x 10^8**	3.65	4.39	8.35	4.78	4.67
**Patient weight (kg)**	74	68.7	60.4	77.5	149

Fabrazyme or agalsidase-β: 1.0 mg/kg/2 weeks (Sanofi-Genzyme Corporation, Cambridge MA)
Replagal or agalsidase-alfa: 0.2 mg/kg/2 weeks (Shire/Takeda, Tokyo, Japan)

Supplementary Table 2. Adverse Events

Event	Number	Grade	1	2	3	4	5	Relation to Study Procedures*	Relation to Protocol Treatment^
Hematological									
Anemia	5	1-3		X		X		Definite	Unrelated
Decreased Erythrocytes	1	1				X		Possible	Unrelated
Decreased hematocrit	2	1				X		Definite/Possible	Unrelated
Febrile Neutropenia	1	3				X		Definite	Unrelated
Decreased Lymphocytes	6	1-4				X		Definite/Possible	Unrelated
Decreased monocytes	2	1				X		Possible	Unrelated
Decreased Neutrophils	9	1-4		X	X	X	X	Definite	Unrelated
Decreased Platelets	6	1-4		X		X		Definite/Possible	Unrelated
Decreased White Blood Cells	5	2-4				X	X	Definite	Unrelated
Lump on left thigh	1	2		X				Possible	Unrelated
Cardiac									
Murmur	1	1		X				Unlikely	Unlikely
Low jugular venous pressure	1	1				X		Unrelated	Unrelated
Palpitations	1	1			X			Possible	Unlikely
Cardiac troponin increased	1	1			X			Possible	Unrelated
Vascular									
Hypertension	1	3				X		Possible	Unrelated
Hypotension	1	2		X				Definite	Unrelated
Thrombophlebitis	1	2					X	Definite	Unrelated
Gastrointestinal									
Abdominal pain	1	1	X					Probable	Unrelated
Constipation	1	1	X					Unrelated	Unrelated
Diarrhea	2	1,2				X		Possible/Probable	N/A, Unrelated
Flatulence	1	1			X			Unlikely	Unlikely
Hemorrhoids	1	2			X			Unlikely	Unrelated
Nausea	5	1,2	X	X	X	X	X	Definite/Possible/ Probable	N/A, Possible/Unlikely/ Unrelated
Oral mucositis	3	1,2			X	X	X	Definite/Possible/ Probable	N/A, Unlikely, Unrelated
Vomiting	4	1,2		X		X	X	Definite/Probable	N/A, Unrelated
Weight gain	1	1					X	Possible	N/A
Weight loss	2	1,2		X				Definite	Unrelated
Neurological									
Dizziness	2	1		X	X			Definite/Possible	Unlikely/Unrelated
Headache	6	1,2	X	X	X	X	X	Definite/Possible/ Probable/Unrelated	N/A, Unrelated
Musculoskeletal									
Pain (Back, hand, rib, hip)	10	1,2			X	X	X	Definite/Probable/ Unrelated	N/A, Unrelated

Skin and Subcutaneous Tissue									
Alopecia	4	1,2	X	X	X	X		Definite/Probable	Unrelated
Erythema multiforme	1	1					X	Definite	Unrelated
Folliculitis	1	1					X	Unrelated	Unrelated
Scratch to leg	1	1			X			Unrelated	Unrelated
Infection									
Catheter-related infection	1	3					X	Definite	Unrelated
Hepatitis	1	1					X	Possible	Unrelated
Bronchial infection	1	2				X		Unrelated	N/A
Respiratory									
Allergic rhinitis	1	2			X			Unrelated	Unrelated
Cough	1	2	X					Possible	Possible
Dyspnea	1	1					X	Definite	N/A
Nosebleed	1	1		X				Unlikely	Unlikely
Hiccups	1	1			X			Possible	Unrelated
Sore throat	1	1		X				Unlikely	Unrelated
Metabolism									
ALT increased	2	1,2			X			Probable	Unrelated
Anorexia	1	3		X				Definite	Unrelated
AST increased	2	1			X			Probable	Unrelated
Dehydration	1	2		X				Probable	Unrelated
Hypomagnesemia	1	1			X			Probable	Unrelated
Hypophosphatemia	1	1		X				Definite	Unrelated
General Disorders									
Citrate reaction	2	2			X			Definite	N/A
Edema (Facial, Limbs)	2	1,2			X	X		Possible/Probable	Unlikely/Unrelated
Fatigue	5	1-3	X	X	X	X	X	Definite/Possible/ Probable	N/A, Unlikely/Unrelated
Fever	1	1			X			Possible	N/A
Vasovagal Episode	1	1	X					Definite	N/A

*Attribution to study procedures in the context of this study refers to all study procedures such as apheresis, bone marrow transplantation procedure, blood draws, insertion of central lines, etc. as well as any protocol-specified drugs used during the procedures such as administration of G-CSF, melphalan and/or plerixafor.

^Attribution to protocol treatment in the context of this study refers to only the gene therapy product or the autologous CD34+ cells expressing alpha-galactosidase A. N/A: Not applicable, as adverse event observed prior to infusion of autologous transduced CD34+ cells.

Severe adverse events are illustrated in red font.

Supplementary Table 3.

Inclusion and Exclusion Criteria from Protocol (v 5.0, January 09, 2018).

Inclusion Criteria

1. Male patients 18–50 years of age at the time of enrollment
2. Diagnosis of Fabry disease as defined by very low (<7.0% of mean) or absent α-galactosidase A (α-gal A) activity in plasma or leukocytes as measured in Dr. Rupar's laboratory at London Health Sciences Centre (screening sample must be sent to Dr. Rupar's lab to determine eligibility for this inclusion criterion)
3. Classic Fabry disease Type I phenotype with GLA genotyping
4. Patients on ET for minimum of 6 months prior to enrollment
5. Eastern Cooperative Oncology Group (ECOG) Performance status of 0 or 1
6. Adequate organ function within 90 days prior to Pre-Mobilization Phase:
   - liver: serum bilirubin < 1.5× upper limit of normal, aspartate aminotransferase/ alanine aminotransferase (AST/ALT) < 3× upper limit of normal;
   - renal: Adequate renal function (estimated Glomerular Filtration Rate (eGFR) by Chronic Kidney Disease-Epidemiology Collaboration (CKD-EPI) formula (Levey 2009) >45ml/min/1.73m^2
   - pulmonary: diffusing capacity of the lung for carbon monoxide (DLCO), forced expiratory volume in 1 second (FEV1), forced vital capacity (FVC) >50% predicted value (corrected for hemoglobin);
   - heart: left ventricular ejection fraction (LVEF) at rest >45%, fractional shortening >0.25%
7. Willing and capable of signing and giving written informed consent in accordance with Research Ethics Board (REB) requirements
8. Willing to comply with all procedures outlined in the study protocol, cooperative with the protocol schedule, able to return for safety evaluation, or otherwise likely to complete the study

9. Willing to abstain from sexual activity or willing to use double-barrier method during sexual intercourse from day of melphalan administration until 12 months follow-up posttransplant. Sexually active male patients will use condoms and in addition, should ask their female partners with child-bearing potential [defined as a sexually mature woman who has not undergone hysterectomy or who has not been naturally postmenopausal for at least 24 consecutive months (i.e., who has had menses any time in the preceding 24 consecutive months)] to use oral, implantable or injectable contraceptives, contraceptive patch, intrauterine device, diaphragm with spermicidal gel when having sexual intercourse.
10. Willing to not donate sperm after receiving melphalan. Sperm banking will be recommended to any patient who would like to father children in the future.

Exclusion Criteria

1. Males with variant Fabry disease.
2. Female gender
3. Use of immunosuppressive agents or any anticoagulant (warfarin, dabigatran, or other oral anticoagulant, and heparin); antiplatelet agents allowed but may need to be withheld in the presence of bleeding or platelet counts $<50x10^9/L$
4. Ongoing ERT-related IAR of moderate-to severe intensity
5. Blood test positive for Hepatitis B Virus (HBV), Hepatitis C Virus (HCV), Human Immunodeficiency Virus (HIV), Human T-lymphotropic Virus 1/2 (HTLV-1/2) or Venereal Disease Research Laboratory (VDRL). Transmissible Disease testing will be done in Pre-Mobilization Phase 2. Patients will only be excluded from the study if positive for the TD tests listed here in this exclusion.

6. Uncontrolled bacterial, viral, or fungal infections
7. Prior malignancies except resected basal cell carcinoma
8. Chronic Kidney Disease (CKD) stage >3A (eGFR by CKD-epi <45 ml/min/1.73m^2)
9. History of heart failure or LVEF <45% or moderate to severe diastolic dysfunction by standard criteria
10. Arrhythmia: bundle branch block, heart block degree II or III, atrial fibrillation, supraventricular tachycardia, ventricular tachycardia, ventricular fibrillation, cardiac arrest, pacemaker, implantable cardiac defibrillator
11. Coronary artery disease with angina, prior myocardial infarction, percutaneous transluminal coronary angioplasty with or without stent, coronary artery bypass graft surgery, moderate to severe valvular heart disease, valve replacement surgery
12. Prior stroke or transient ischemic attack (TIA); or stroke on prior brain imaging
13. Uncontrolled hypertension ≥150/90
14. Diabetes mellitus
15. Advanced liver disease, liver failure, cirrhosis
16. Immune deficiency state
17. Moderate-to-severe chronic obstructive pulmonary disease (COPD)
18. Any hematological condition with white blood cells (WBC) <3.0 x10^9/L, platelet count <100 x10^9/L, and/or hemoglobin <100 g/L
19. Prior BMT or organ transplant
20. Any condition that would preclude use of melphalan
21. Use of a drug with cytotoxic or immunosuppressive effect within 60 days of trial entry
22. Uncontrolled psychiatric disorder

23. Active chronic infection
24. Prior tuberculosis
25. Any other serious concurrent disease in the investigator's opinion, that could affect study endpoints
26. Cognitive impairment that would prevent informed consent
27. Use of an investigational drug within 30 days of stem cell transplant

Supplementary Table 4. Criteria For Patients To Undergo An ET Withdrawal

- The patient continues to make his own α-galactosidase A (α-gal A) enzyme at 6 months post-transplant as determined in the study reference laboratory at a level deemed acceptable by the investigator.
- The patient is deemed medically stable by their study physician.
- The patient provides consent for withdrawal of ET following discussion with their study physician, and this has been documented by the physician and/or nurse that it was discussed with the patient and they are in agreement.
- The Clinical Trials Steering Committee unanimously agree and provide written approval that ET may be stopped for the patient after reviewing all relevant data and literature, and evaluating the risks vs benefits to the patient.

Should any of the patients show any safety concerns after stopping ET, ET may re-start as per investigators discretion.

Supplementary Table 5. Primers Utilized for Quantitative Polymerase Chain Reactions

Primer/Probe name:	Product:	Sequence:	Supplier:
WPRE Forward	116bp	5'-TCCTGGTTGCTGTCTCTTTATG-3'	Custom Sigma Aldrich order
WPRE Reverse	116bp	5'-TGACAGGTGGTGGCAATG-3'	Custom Sigma Aldrich order
WPRE TaqMan Probe	116bp	5' VIC-TGCTGACGCAACCCCCACTGGT-TAMRA 3'	Custom Thermo Fisher Scientific order, Catalog # 450025
Human β-actin TaqMan assay	139bp	Assay ID: Hs03023880_g1	Thermo Fisher Scientific, Catalog #4331182

Supplementary Note 1

The current version of the study protocol *"Clinical Pilot Study of Autologous Stem Cell Transplantation of CD34+ Cells Engineered to Express Alpha-Galactosidase A in Patients with Fabry Disease"* is appended below.

CLINICAL STUDY PROTOCOL

Clinical Pilot Study of Autologous Stem Cell Transplantation of $CD34^+$ Cells Engineered to Express Alpha-Galactosidase A in Patients with Fabry Disease

Clinical Trial Protocol No: Ozmosis Study No. OZM-074

Protocol Version #: 5.0

Protocol Date: 09-JAN-2018

Phase of Study: I

Sponsor: University Health Network

Sponsor Address: 610 University Avenue

Toronto, ON M5G 2M9

Protocol History

Original:	Version #1.0; dated 23-MAR-2016
Amendment#1:	Version #2.0; dated 25-OCT-2016
Amendment#2:	Version #3.0; dated 06-FEB-2017
Amendment #3:	Version #4.0; dated 16-AUG-2017
Amendment #4:	Version #5.0; dated 09-JAN-2018

Sponsor's Medical Monitor:

Dr. Daniel Fowler
National Institutes of Health
National Cancer Institute
Experimental Transplantation and Immunology Branch
Head, Cytokine Biology Section Building 10, CRC,
3-EAST Labs, 3-3330 Bethesda, MD 20892 USA
Tel: 301-640-1818
Email: dhfowler@helix.nih.gov

Lead Clinical Investigators:

Dr. Ronan Foley
Juravinski Cancer Centre
711 Concession St. Hamilton, ON L8V1C3
Tel: 905-527-4322 ext 42074
Fax: 905-575-2553
Email: foleyr@hhsc.ca

Dr. Michael West
5090 ACC QE II Health Sciences Centre,
5820 University Ave, Halifax NS B3H 1V8
Tel: 902 473-4023
Mobile: 902 488-3785
Fax: 902 473-2675
Email: mlwest@dal.ca

Dr. Aneal Khan
Alberta Children's Hospital, University of Calgary
3rd Floor Metabolic Clinic
2888 Shaganappi Trail NW
Calgary, Alberta.
Tel: 403 955 7587
Fax: 403 955 3091
Email: khaa@ucalgary.ca

Dr. Armand Keating
Suite 5-303 Princess Margaret Cancer Centre
610 University Avenue, M5G 2M9, ON
Tel: 416-946-4595
Fax: 416-946-4530
Email: armand.keating@uhn.ca

Co-Investigators: Dr. Stephen Couban
Division of Hematology
QEII - Bethune Building
Suite 430 Bethune Building
1276 South Park Street
Halifax, NS B3H 2Y9
Tel: 902-473-8562
Fax: 902-473-4600
Email: Stephen.couban@nshealth.ca

Dr. Andrew Daly
Tom Baker Cancer Centre
1331 29 Street NW
Calgary, AB T2N 4N2
Tel: 403-521-3706
Email: Andrew.Daly@albertahealthservices.ca

Dr. Chantal Morel
UHN and MSH 60 Murray Street Box 34 3rd Floor Room
400 Toronto, ON, M5T3L9
Tel: 416-586-4800 ext4224 (direct), ext 4232 (admin)
Fax: 416-619-5523
Email: Chantal.morel@uhn.ca

Lab Investigators: Dr. Tony Rupar
London Health Sciences Centre
Victoria Hospital-Westminster Tower
Room E3-208
800 Commissioners Road East
London, Ontario, N6H 5W9
Tel: 519-685-8500 x 71558
Fax: 519-858-1063
Email: Tony.Rupar@lhsc.on.ca

Dr. Christiane Auray-Blais
Division of Medical Genetics, Lab 1419
CIUSSS-CHUS Hospital Fleurimont
3001, 12th Avenue North
Sherbrooke, Qc Canada J1H 5N4
Tel: 819 346-1110 ext. 14706
Fax: 819-564-5217
Pager: 819-348-3024
Email: christiane.auray-blais@USherbrooke.ca

Project PI/Sponsor:

Dr. Jeffrey Medin
University Health Network
Tel: 414-955-4118
Fax: 414-955-6043
Email: jmedin@mcw.edu

Clinical Trial Management Company/Clinical Trials Manager:

Rupi Mangat
Ozmosis Research Inc.
65 Queen St West, Suite 2020,
Toronto, ON M5H 2M5
Direct Line: 416-634-8308
Main Line: 416-634-8300
Fax: 416-598-4382
E-mail: rupi.mangat@ozmosisresearch.ca

Sponsor's Agreement to Protocol Version #5.0, 09-JAN-2018

Name of Authorized Personnel (Print) ________________________________

Title of Authorized Personnel (Print) ________________________________

Signature of Authorized Personnel: ________________________________

Date of Approval: ________________________________
DD-MMM-YYYY

SYNOPSIS

**Study Title:**	Clinical Pilot Study of Autologous Stem Cell Transplantation of $CD34^+$ Cells Engineered to Express Alpha-Galactosidase A in Patients with Fabry Disease
**Primary Objectives:**	To determine the overall safety and toxicity of autologous stem cell transplantation (ASCT) with mobilized $CD34^+$ cells transduced with the LV vector containing human codon-optimized α-gal A (aka LV/AGA) in adult males with FD
**Secondary Objectives:**	To obtain preliminary evidence of the efficacy of the procedure in patients with FD by assessing the following laboratory parameters:<br><br>a. levels of α-gal A activity in plasma, peripheral blood leukocytes (PBL), and bone marrow mononuclear cells (BMMC)<br>b. levels of globotriaosylceramide (Gb_3) in plasma and urine<br>c. levels of globotriaosylsphingosine (lyso-Gb_3) and related analogues in plasma and urine<br>d. presence and persistence of marked cells with human codon-optimized α-gal A in peripheral blood<br>e. blood (plasma, serum) and urine samples will be obtained for future determination of biomarkers or other metabolites as needed<br>f. measure the transduction efficiency of hematopoietic/progenitor stem cells
**Study Design:**	This is a multi-centre, non-randomized, open-label, prospective pilot study of a novel stem cell gene therapy protocol.<br><br>The study will be conducted in four phases: Phase 1 - Screening, Phase 2 – Preparing Final Product phase, Phase 3 -Treatment phase and Phase 4 - Post-Treatment Follow Up phase. Refer to Figure 1 and Study Calendar (Section 5).<br><br>Safety review meetings will take place to review each patient's safety data after each patient receives the transplant and completes the 1 month post-transplant follow up visit. Only when it is deemed safe (no protocol-treatment related SAEs or significant safety concerns) can the next patient start treatment phase of the study.
**Duration:**	Duration of patients on study (from Pre-Mobilization phase to one month follow up) is approximately 3 months. Patients will be followed for total of 5 years

	after receiving transplant.
**Planned Total Sample Size:**	Up to 6 adult male patients with Fabry Disease
**Test Administration:**	Autologous transduced cells that have been approved by Health Canada (Certificate of Analysis approved) will be re-infused into eligible patients.
**Inclusion/Exclusion Criteria:**	**Inclusion Criteria**<br><br>1. Male patients 18–50 years of age at the time of enrollment<br>2. Diagnosis of FD as defined by very low (<7.0% of mean) or absent α-gal A activity in plasma or leukocytes as measured in Dr. Rupar's laboratory at London Health Sciences Centre (screening sample must be sent to Dr. Rupar's lab to determine eligibility for this inclusion criterion)<br>3. Classic FD Type I phenotype with GLA genotyping<br>4. Patients on ERT for minimum of 6 months prior to enrollment<br>5. Eastern Cooperative Oncology Group (ECOG) Performance status of 0 or 1<br>6. Adequate organ function within 90 days prior to Pre-Mobilization Phase:<br>-liver: serum bilirubin < 1.5× upper limit of normal, aspartate aminotransferase/ alanine aminotransferase (AST/ALT) < 3× upper limit of normal;<br>-renal: Adequate renal function (estimated GFR (eGFR) by CKD-epi formula[1] >45 ml/min/1.73m^2<br>-pulmonary: diffusing capacity of the lung for carbon monoxide (DLCO), forced expiratory volume in 1 second (FEV1), forced vital capacity (FVC) >50% predicted value (corrected for hemoglobin);<br>-heart: left ventricular ejection fraction (LVEF) at rest >45%, fractional shortening >0.25%<br>7. Willing and capable of signing and giving written informed consent in accordance with Research Ethics Board (REB) requirements<br>8. Willing to comply with all procedures outlined in the study protocol, cooperative with the protocol

	schedule, able to return for safety evaluation, or otherwise likely to complete the study<br>9. Willing to abstain from sexual activity or willing to use double-barrier method during sexual intercourse from day of Melphalan administration until 12 months follow-up post-transplant. Sexually active male patients will use condoms and in addition, should ask their female partners with child-bearing potential [defined as a sexually mature woman who has not undergone hysterectomy or who has not been naturally postmenopausal for at least 24 consecutive months (i.e. who has had menses any time in the preceding 24 consecutive months)] to use oral, implantable or injectable contraceptives, contraceptive patch, intrauterine device, diaphragm with spermicidal gel when having sexual intercourse. Refer to Section 6.9.<br>10. Willing to not donate sperm after receiving Melphalan. Sperm banking will be recommended to any patient who would like to father children in the future.<br><br>**Exclusion Criteria**<br>1. Males with variant Fabry Disease.<br>2. Female gender<br>3. Use of immunosuppressive agents or any anticoagulant (warfarin, dabigatran, or other oral anticoagulant, and heparin); antiplatelet agents allowed but may need to be withheld in the presence of bleeding or platelet counts <$50x10^9$/L<br>4. Ongoing ERT-related IAR of moderate-to-severe intensity<br>5. Blood test positive for HBV, HCV, HIV, HTLV-1/2 or VDRL (Transmissible Disease testing will be done in Pre-Mobilization Phase 2 – see section 5.1 for full panel of TD tests. Patients will only be excluded from the study if positive for the TD tests listed here in this exclusion).<br>6. Uncontrolled bacterial, viral, or fungal infections<br>7. Prior malignancies except resected basal cell carcinoma

	8. Chronic Kidney Disease (CKD) stage >3A (eGFR by CKD-epi <45 mL/min/$1.73m^2$)<br>9. History of heart failure or LVEF <45% or moderate to severe diastolic dysfunction by standard criteria<br>10. Arrhythmia: bundle branch block, heart block degree II or III, atrial fibrillation, supraventricular tachycardia, ventricular tachycardia, ventricular fibrillation, cardiac arrest, pacemaker, implantable cardiac defibrillator<br>11. Coronary artery disease with angina, prior myocardial infarction, percutaneous transluminal coronary angioplasty with or without stent, coronary artery bypass graft surgery, moderate to severe valvular heart disease, valve replacement surgery<br>12. Prior stroke or transient ischemic attack (TIA); or stroke on prior brain imaging<br>13. Uncontrolled hypertension ≥150/90<br>14. Diabetes mellitus<br>15. Advanced liver disease, liver failure, cirrhosis<br>16. Immune deficiency state<br>17. Moderate-to-severe chronic obstructive pulmonary disease (COPD)<br>18. Any hematological condition with white blood cells (WBC) <3.0 $x10^9$/L, platelet count <100 $x10^9$/L, and/or hemoglobin <100 g/L<br>19. Prior BMT or organ transplant<br>20. Any condition that would preclude use of Melphalan<br>21. Use of a drug with cytotoxic or immunosuppressive effect within 60 days of trial entry<br>22. Uncontrolled psychiatric disorder<br>23. Active chronic infection<br>24. Prior tuberculosis<br>25. Any other serious concurrent disease in the investigator's opinion, that could affect study endpoints<br>26. Cognitive impairment that would prevent informed consent

	27. Use of an investigational drug within 30 days of SCT
**Phase 1: Screening Assessments**	• Written Informed Consent<br>• Inclusion/Exclusion Criteria<br>• Medical History (History of FD)<br>• Physical Examination<br>• Weight & ECOG<br>• Vital Signs<br>• CBC, differential<br>• Electrolytes, urea/BUN, creatinine<br>• Serum Chemistry<br>• INR, PTT<br>• Serum Troponin, CK<br>• 24hr urine creatinine, protein<br>• Urinalysis<br>• 24-hour Holter Monitoring<br>• 12-lead ECG<br>• ECHO or MUGA<br>• Brain MRI (optional)<br>• Cardiac MRI (optional)<br>• Pulmonary Function Tests<br>• T/B cell count (optional)<br>• ERT administration<br>• Plasma α-Gal A activity<br>• α-Gal A activity in leukocytes<br>• Plasma Gb3 & isoforms & analogues<br>• Plasma lyso-Gb3 and related analogues<br>• Urine lyso-Gb3 and related analogues<br>• Urine Gb3 & isoforms and analogues<br>• Anti-agalsidase antibodies assays<br>• Concomitant Medications<br>• Baseline Symptoms
**Phase 2: Preparing Final Product – Assessments:**	• Vital Signs<br>• Transmissible Disease Testing<br>• ERT administration<br>• Plasma α-Gal A activity<br>• α-Gal A activity in leukocytes<br>• Plasma Gb3 & isoforms & analogues<br>• Plasma lyso-Gb3 and related analogues<br>• Urine lyso-Gb3 and related analogues<br>• Anti-agalsidase antibodies assays<br>• Urine Gb3 & isoforms and analogues

	• Optional correlatives: urine metabolite assays<br>• Concomitant Medications<br>• Baseline Symptoms
**Phase 3: Treatment Assessments**	• Vital Signs<br>• Physical Examination<br>• Weight & ECOG<br>• CBC, Differential<br>• Electrolytes, urea/BUN, creatinine<br>• Serum Chemistry<br>• INR, PTT<br>• Group and screen<br>• Neupogen (filgrastim) injection (Mobilization and Transplant phase)<br>• Harvest stem cells<br>• Pre-absolute CD34 count/µL<br>• Flow Cytometry<br>• Serum Troponin, CK<br>• 24hr urine creatinine, protein<br>• Albumin-to-creatinine ratio<br>• 24-hr Holter Monitoring<br>• Urinalysis<br>• 12-lead ECG<br>• ECHO or MUGA<br>• Cardiac MRI<br>• Chest X-ray (or CT chest)<br>• T/B cell count<br>• Melphalan administration<br>• Infusion of transduced stem cells<br>• Plasma α-Gal A activity<br>• α-Gal A activity in leukocytes<br>• Bone marrow aspirate<br>• Plasma Gb3 & isoforms & analogues<br>• Plasma lyso-Gb3 and related analogues<br>• Urine lyso-Gb3 and related analogues<br>• Urine Gb3 & isoforms and analogues<br>• Optional correlatives: urine metabolite assays<br>• Concomitant Medications<br>• Adverse Events
**Phase 4: Post-Treatment Follow up Assessments**	• Vital Signs<br>• Physical Examination<br>• Weight & ECOG<br>• CBC, Differential

	• T/B cell count<br>• Electrolytes, urea/BUN, creatinine<br>• Serum Chemistry<br>• 24hr urine creatinine, protein<br>• Plasma α-Gal A activity<br>• α-Gal A activity in leukocytes<br>• Plasma Gb3 & isoforms & analogues<br>• Plasma lyso-Gb3 and related analogues<br>• Urine lyso-Gb3 and related analogues<br>• Urine Gb3 & isoforms and analogues<br>• Anti-agalsidase antibodies assays<br>• Urinalysis<br>• Optional correlatives: urine metabolite assays<br>• Chest X-ray (or CT chest if CT done at screening)<br>• 12-lead ECG<br>• 24-hr Holter Monitoring<br>• ECHO or MUGA<br>• Brain MRI<br>• Cardiac MRI<br>• Pulmonary Function Tests<br>• Concomitant Medications<br>• Adverse Events
**Correlative Assessments:**	**Mandatory Correlatives:**<br><br>For each patient, urine and plasma specimens for the analysis of Gb3 isoforms/analogues and lyso-Gb3 and analogues will be collected as per the Study Calendar (Refer to Section 5).<br><br>**Optional Correlatives:**<br><br>We will use leftover urine samples from the mandatory studies sent to Sherbrooke lab for these optional studies (Galabiosylceramide (Ga_2) isoforms/analogues).
**Response:**	**Assessment of Functional Efficacy Measurements**<br><br>The following laboratory measures of efficacy will be determined:<br><br>• Increase in α-gal A enzyme activity within the plasma, leukocytes, and BM<br>• Reduction of Gb_3 in plasma and urine

	• Reduction of lyso-Gb_3 and related analogues in plasma and urine<br>• Persistence of LV-transduced cells as measured by quantitative (q)PCR<br>• Measure the transduction efficiency of hematopoietic stem/progenitor cells
**Safety Variables & Analysis:**	Adverse events will use the descriptions and grading scales found in the revised NCI Common Terminology Criteria for Adverse Events (CTCAE). This study will utilize the CTCAE Version 4.03 for adverse event reporting.
**Statistical Analysis:**	Tabulations and descriptive statistics will be employed in the analysis of primary and secondary objectives. Adverse events will be tabulated by National Cancer Institute of Canada Clinical Trials Group (NCIC CTG) Expanded Common Toxicity Criteria version 4.03. Additional summary tables will be generated for patients with serious adverse events and patients with related adverse events. Statistical analysis of laboratory observations will include comparison of means or medians by Paired T test, the Wilcoxon signed rank test and mixed model as necessary. Significance will be set at a 0.05 level. As the expected number of subjects is only up to 6, statistical analysis will be limited in power and the nature of the analysis is exploratory.

TABLE OF CONTENTS

SYNOPSIS ... 6
Assessment of Functional Efficacy Measurements ... 12
LIST OF ABBREVIATIONS ... 17
TREATMENT SCHEMA ... 20
1. OBJECTIVES ... 21
1.1. Primary Objective ... 21
1.2. Secondary Objectives ... 21
2. BACKGROUND AND RATIONALE ... 21
2.1. Fabry Disease and Current Treatment ... 21
2.2. Recombinant LV-based Gene Therapy Clinical Trials ... 22
2.3. Pre-Clinical Studies of LV-based Gene Therapy for Fabry Disease ... 23
2.4. Recombinant Lentiviral Vector Design ... 24
2.5. Study Rationale ... 24
3. TRIAL DESIGN ... 26
4. PATIENT POPULATION ... 27
4.1. Inclusion Criteria ... 27
4.2. Exclusion Criteria ... 28
4.3. Patient Registration and Enrollment ... 29
5. STUDY CALENDAR ... 32
5.1. Footnotes ... 37
6. TREATMENT PLAN ... 40
6.1. Enrollment Procedures and Safety Review Meeting ... 40
6.1.1. Safety Stopping Rules ... 40
6.1.2. Clinical Trial Steering Committee (CTSC) ... 40
6.1.3. Data Safety Monitoring Committee (DSMC) ... 41
6.2. Data Required for Safety Review Meeting ... 41
6.3. Cardiology Review ... 41
6.4. Study Phases ... 42
6.4.1. Phase 1: Screening (within 90 days prior to Pre-Mobilization phase) ... 42
6.4.2. Phase 2: Preparing for Final Product ... 42
6.4.2.1. Stage 1: Pre-Mobilization (28 days lead-in prior to Mobilization phase) ... 42
Enzyme Replacement Therapy (ERT) ... 42
6.4.2.2. Stage 2: Mobilization (Days 1 to 6 or 7: Day 7 is Optional. If insufficient number of cells are collected on Days 5 and/or 6, then there may be an additional collection day on day 7) ... 43
6.4.2.3. Stage 3: Leukapheresis (Days 3, 4, 5, 6 or 7) ... 43
6.4.2.4. Stage 4: CD34+ Selection/Enrichment (at Juravinski Health Sciences Centre in Hamilton) ... 44
6.4.2.5. Stage 5: Transduction and Cryopreservation (at Orsino Cell Processing Facility in Princess Margaret Hospital) ... 44
6.4.2.6. Stage 6: Approval of Certificate of Analysis (CoA) from Health Canada for Release of Product ... 45
6.4.3. Phase 3: Treatment Phase - Transplant (Days -2 to +12) ... 45
A) Day -2 ... 45
B) Day -1: Melphalan Administration ... 45
C) Day 0: Health Canada Approved Transduced Autologous CD34^{+} Cell Product for Infusion ... 45
D) Day 5: Neupogen® (filgrastim) Injection ... 46
Re-infusion of Back-up Autograft of Non-Transduced, Non-CD34+ Selected Cells ... 46
6.4.4. Phase 4: Post-Treatment Follow Up for 5 Years at Regional FD Centre ... 46
6.4.4.1. Enzyme Replacement Therapy Resumes ... 46
6.4.4.2. Enzyme Replacement Therapy may be stopped after 6 months post-transplant ... 47
6.4.4.3. Post-Transplant Management ... 48
6.5. Treatment Delays ... 48
6.6. Concomitant Medications ... 48
6.6.1. Supportive Therapy and Permitted Concomitant Medications ... 48
6.6.2. Prohibited Concomitant Medications ... 48
6.7. Premature Withdrawal/Discontinuation of Study Participants ... 48
6.8. Subjects Compliance and Dropout ... 49

6.9. Contraception 49
7. RISKS VS. BENEFITS 50
7.1. Benefits 50
7.2. Risks Due to Stem Cell Mobilization 50
G-CSF (Refer to Neupogen Product Monograph for details on risks) 50
7.3. Risks of Stem Cell Collection/Apheresis 50
7.4. Risks of ex vivo Cell Transductions 50
7.5. Intensive Therapy and Stem Cell Infusion 50
7.6. Lentivirus Vectors (LVs) 51
7.7. Anti- α-gal A Antibody Development 51
7.8. Enzyme Replacement Therapy 52
7.8.1. IARs (Infusion-Associated Reaction) 53
8. STUDY INTERVENTION / INVESTIGATIONAL PRODUCT 54
8.1. Investigational Product 54
Preparation, Transport and Labelling 54
8.2. 54
8.3. Drug Product Accountability 55
8.4. Stability 55
8.5. Drug Product Administration 55
9. CRITERIA FOR MEASUREMENT OF STUDY OUTCOMES AND STUDY ENDPOINTS 55
9.1. Primary Endpoint 55
9.2. Secondary Endpoints: Assessment of Functional Efficacy Measurements 55
9.2.1. Increase in α-Galactosidase A Activity 55
9.2.2. Globotriaosylceramide (Gb3) levels 56
9.2.3. Levels of Globotriaosylsphingosine (lyso-Gb3) and Related Analogues 56
9.2.4. Methyl-cellulose PCR Assay 56
9.3. Secondary Safety Measurements 56
9.3.1. Generation of Replication Competent Lentiviruses (RCL) 56
9.3.2. Vector copy number & persistence by quantitative real-time PCR 57
9.3.3. Anti-agalsidase A Antibody Level Determination 57
9.4. Correlative Studies 57
9.4.1. Mandatory Correlative Studies 57
9.4.1.1. Analysis of Gb_3 isoforms/analogues in urine and plasma 57
9.4.1.2. Analysis of lyso-Gb_3 and analogues in plasma 57
9.4.2. Optional Correlative Studies 57
9.4.2.1. Optional metabolites studies in urine 57
9.4.3. Optional Research Studies 58
9.4.3.1. Alpha Gal –A Activity in Optional Skin Biopsy 58
9.4.3.2. Examining Clonality in Optional Blood Samples 58
9.4.3.3. Examining Podocytes in Optional Urine Samples 58
10. SAFETY AND REPORTING REQUIREMENTS 59
10.1. Adverse Event 59
10.2. Adverse Event Documentation 59
10.3. Serious Adverse Event 59
10.4. Reporting Serious Adverse Events 61
10.5. Procedure for Expedited Reporting 61
10.6. Adverse and Serious Adverse Events and Reporting Period 62
10.7. Relationship 63
11. STATISTICAL CONSIDERATIONS 63
11.1. Study Design and Justification of Sample Size 63
11.2. Study Population 64
11.3. Statistical Analysis Methods 64
11.4. Evaluation of Safety 64
11.5. Evaluation of Response 64
12. ETHICS 65
12.1. Informed Consent 65

12.2. Ethics Board Approval....65
13. DOCUMENTATION, RECORD ACCESS AND MAINTENANCE OF STUDY RECORDS 65
13.1. Documentation of Subject's Participation 66
13.2. Regulatory Requirements 66
13.3. Subject Confidentiality and Access to Source Data/Documents 66
13.4. Confidentiality of the Study....66
13.5. Registration of Clinical Trial 67
13.6. Data Reporting and Data Management....67
13.7. Case Report Forms 67
13.8. Maintenance of Study Records 67
14. QUALITY ASSURANCE AND QUALITY CONTROL 68
14.1. On Site Monitoring / Auditing....68
15. ADMINISTRATIVE PROCEDURES....68
15.1. Amendments to the Protocol....68
15.2. Protocol Deviations and Violations 68
15.3. Premature Discontinuation of the Study 68
16. LEGAL ASPECTS 69
16.1. Responsibilities of the Investigator....69
16.2. Publication Policies 69
17. Appendix I: ECOG Performance Status....70
18. APPENDIX II: Common Terminology Criteria for Adverse Events 71
19. REFERENCES....72

LIST OF ABBREVIATIONS

1.	α-gal A	α-galactosidase A
2.	ABMT	autologous bone marrow transplant
3.	ACDase	acid ceramidase
4.	AE	adverse event
5.	ALD	Adrenoleukodystrophy
6.	ALP	alkaline phosphatase
7.	ALT	alanine aminotransferase
8.	ANC	absolute neutrophil count
9.	APC	antigen presenting cell
10.	ASCT	autologous stem cell transplant
11.	AST	aspartate aminotransferase
12.	BM	bone marrow
13.	BMMC	bone marrow mononuclear cells
14.	BMT	bone marrow transplant
15.	BUN	blood urea nitrogen
16.	CBC	complete blood count
17.	cDNA	complementary DNA
18.	CFDI	Canadian Fabry Disease Initiative
19.	CK	creatinine kinase
20.	CKD	Chronic Kidney Disease
21.	CMV	Cytomegalovirus
22.	COPD	Chronic Obstructive Pulmonary Disease
23.	CRF	case report form
24.	CRO	Contract Research Organization
25.	CT	computed tomography
26.	CTCAE	Common Terminology Criteria for Adverse Events
27.	CXR	chest x-ray
28.	DLCO	diffusing capacity of the lung for carbon monoxide
29.	DNA	deoxyribonucleic acid
30.	DSMC	Data Safety Monitoring Committee
31.	ECG	electrocardiogram
32.	ECOG	Eastern Cooperative Oncology Group
33.	eGFR	estimated glomerular filtration rate
34.	ELISA	enzyme-linked immunosorbent assay
35.	ERT	enzyme replacement therapy
36.	FACS	fluorescence-activated cell sorter (flow cytometric analysis)
37.	FACT	Foundation for Accreditation of Cell Therapy
38.	FD	Fabry disease
39.	FEV1	forced expiratory volume in 1 second
40.	Flt3-L	Flt3 ligand
41.	FVC	forced vital capacity
42.	Gb_3	globotriaosylceramide
43.	GCP	Good Clinical Practice
44.	G-CSF	granulocyte-colony stimulating factor
45.	GMP	Good Manufacturing Practice

46.	GSL	glycosphingolipid
47.	HBV	Hepatitis B Virus
48.	HCV	Hepatitis C Virus
49.	HIV	Human Immunodeficiency Virus
50.	HPLC	high performance liquid chromatography
51.	HSPC	hematopoietic stem/progenitor cells
52.	HSV	Herpes Simplex Virus
53.	HTLV-1	Human T-cell Lymphotropic Virus type 1
54.	IAR	infusion-associated reaction
55.	ICF	Informed Consent Form
56.	ICH	International Conference on Harmonization
57.	Ig	immunoglobulin
58.	IL-3	interleukin-3
59.	INR	international ratio
60.	IUVPF	Indiana University Vector Production Facility
61.	I.V.	intravenous
62.	LDH	lactate dehydrogenase
63.	LSD	lysosomal storage disorder
64.	LTR	long-terminal repeat
65.	LV	lentivirus; lentivector
66.	LV/AGA	LV/hum.co.α-gal A
67.	LVEF	left ventricular ejection fraction
68.	Lyso-Gb_3	globotriaosylsphingosine (deacylated globotriaosylceramide)
69.	MLD	Metachromatic Leukodystrophy
70.	MRI	magnetic resonance imaging
71.	MUGA	multi-gated acquisition scan
72.	NCIC	National Cancer Institute of Canada
73.	NCIC CTG	National Cancer Institute of Canada Clinical Trials Group
74.	NHP	non-human primate
75.	PBL	peripheral blood leukocytes
76.	PBMC	peripheral blood mononuclear cells
77.	PBPC	peripheral blood progenitor cells
78.	PBS	phosphate buffered saline
79.	PBSCT	peripheral blood stem cell transplantation
80.	PCR	polymerase chain reaction
81.	PFT	pulmonary function tests
82.	PI	Principal Investigator
83.	p.o.	per os (take orally)
84.	PSOCTF	Philip S. Orsino Cell Therapy Facility
85.	PTT	partial thromboplastin time
86.	QEII	Queen Elizabeth II Hospital, Halifax
87.	QI	Qualified Investigator
88.	qPCR	quantitative PCR
89.	RCL	replication competent lentivirus
90.	REB	Research Ethics Board

91.	SAE	serious adverse event
92.	s.c.	subcutaneous
93.	SCF	stem cell factor
94.	SIN	self-inactivating
95.	s.l.	sublingual
96.	SOP	Standard Operating Procedure
97.	SUSAR	suspected, unexpected, and serious adverse reactions
98.	TIA	transient ischemic attack
99.	TLC	total lung capacity
100.	TPO	thrombopoietin
101.	tRNA	transfer RNA
102.	UHN	University Health Network
103.	VDRL	Venereal Disease Research Laboratory
104.	VSV-g	Vesicular Stomatitis Virus glycoprotein
105.	WBC	white blood cells
106.	WPRE	Woodchuck hepatitis virus post-transcriptional regulatory element

TREATMENT SCHEMA

Patients provide written informed consent (and optional correlatives consent)

⬇

Phase 1: Screening Phase (within 90 d prior to Pre-Mobilization Phase)
Adult male patients with a diagnosis of Fabry Disease and α-gal A deficiency screened and eligibility confirmed at each site. Eligible patients registered onto trial.

⬇

Phase 2: Pre-Mobilization (28 days prior to Mobilization Phase)
ERT administration every 14 days until D28 (or at least 30 days prior to ASCT) and establishment of baseline α-Gal A activity

⬇

Phase 2: – Mobilization (D1 to 6 or 7)
At each Site, mobilize with Neupogen® and harvest autologous PBPC (min. 10.0 x 10^6CD34 cells/kg). Plerixafor may be used if <7.5x10^6CD34 cells/kg obtained on first day of collection.
(back-up, non-selected PBPC maintained – min. 2.5 x10^6 CD34 cells/kg)
Ship remaining sample (min. 7.5x10^6 CD34 cells/kg) immediately to Juravinski Cancer Centre

⬇

$CD34^+$ selection at Juravinski Cancer Centre, Hamilton
Ship $CD34^+$ selected cells to Philip S. Orsino Cell Therapy Facility, PMH Toronto

⬇

At PMH, transduction with LV- hum.co.α-gal A (i.e. LV/AGA), cryopreservation
Certificate of Analysis Approved by Health Canada
Ship autologous transduced cells back to Site

⬇

Phase 3: Treatment Phase – Transplant (D-2 to +12)
Enrolled patient will receive Melphalan 100 mg/m^2 at the Regional Stem Cell Transplant Centre. Thaw and re-infusion of autologous transduced cells.

⬇

Phase 4: Post-Treatment Follow up Phase to 5 years
Follow-up at regional hospital with Designated Co-Investigator. After 1 month of scheduled follow-up post-transplant, safety review of patient data will be conducted.
If no protocol treatment-related Serious Adverse Events or no significant safety concerns, enroll next patient to a maximum of six patients (safety review will be done after each patient completes 1 month follow up post-transplant before next patient can start treatment phase of study)

⬇

Follow up in regional FD centre for 5 years
Resume ERT at 1 month post-transplant. Patient may stop ERT after 6 months post-transplant if ERT stopping critieria are met.

1. OBJECTIVES

1.1. Primary Objective

To determine the overall safety and toxicity of autologous stem cell transplantation (ASCT) with mobilized $CD34^+$ cells transduced with the LV vector containing human codon-optimized α-gal A (aka LV/AGA) in adult males with FD

1.2. Secondary Objectives

To obtain preliminary evidence of the efficacy of the procedure in patients with FD by assessing the following laboratory parameters:

a) levels of α-gal A activity in plasma, peripheral blood leukocytes (PBL), and bone marrow mononuclear cells (BMMC)
b) levels of globotriaosylceramide (Gb_3) in plasma and urine
c) levels of globotriaosylsphingosine (lyso-Gb_3) and related analogues in plasma and urine
d) presence and persistence of marked cells with human codon-optimized α-gal A in peripheral blood
e) blood (plasma, serum) and urine samples will be obtained for future determination of biomarkers or other metabolites as needed
f) measure the transduction efficiency of hematopoietic stem/progenitor cells

2. BACKGROUND AND RATIONALE

2.1. Fabry Disease and Current Treatment

Fabry disease is an X-linked Lysosomal Storage Disorder (LSD) caused by a deficiency in the activity of α-gal A. As a result, glycosphingolipids (GSL) with terminal α-galactosyl moieties accumulate in virtually all cells and tissues, mainly as Gb_3[2]. Males die in mid-life of cardiovascular disease, cerebrovascular disease, or renal failure. Heterozygote females are not just carriers but can be affected as well. Women show disease heterogeneity from being asymptomatic to manifesting severe organ failure with premature strokes. Variant late-onset phenotypes of FD that are associated with a few specific genotypes also exist, with disease limited mainly to cardiac or renal involvement associated with increased residual α-gal A activity.

According to recent data on a cohort of 1765 males and females from the Fabry Registry, signs (renal disease, cornea verticillata, and angiokeratomas) and symptoms (neurological pain, diarrhea, and abdominal pain) present at a median age of onset of 9 years in hemizygous males and 13 years in heterozygous females, respectively[3]. The median onset of cardiovascular and cerebrovascular events occurred in that cohort in the early forties for males and in the mid-forties for females. The median age at occurrence of renal progression failure was 38 years for both sexes[3].

Treatment strategies for FD have traditionally focused on management of symptoms, until the availability of specific therapy with Enzyme Replacement Therapy (ERT) in 2000[4,5]. ERT for FD relies on a phenomenon common to many lysosomal hydrolases called metabolic cooperativity or cross-correction, wherein enzyme can be taken up into many cell types through mannose-6-phosphate receptor-mediated endocytosis. There are currently two preparation of α-gal A for clinical ERT use: agalsidase alfa (Replagal™, Shire Inc.) and agalsidase beta (Fabrazyme®, Genzyme, a Sanofi Company). Both preparations are used in Fabry clinics in Canada. As the current version of agalsidase alfa is unlicensed in Canada, patients are currently receiving this drug in Canada through a clinical trial but it is anticipated that marketed approval for Replagal in Canada will occur in the near future. Agalsidase beta, however, is approved by Health Canada. Other therapeutic paradigms involving small molecules have also been proposed for treating FD, including substrate reduction therapy[6] and molecular pharmacologic chaperone therapy[7]. While short-term studies are now available for chaperone therapy in Fabry disease, there are no outcome data on substrate reduction therapy. However, these agents are not licensed in any jurisdiction to date and, like ERT, they do not address the core molecular defect of the disorder.

The high cost of lifelong therapy with ERT is another major concern. The yearly cost of ERT for a patient weighing 70 kg is currently about $250,000. This suggests that lifelong ERT cannot be cost-effective even if it completely prevents the medical complications of FD and extends life for 30 years or more[8]. Given the early onset of symptoms in FD, the progressive nature of this disease, and the fact that ERT has only really been shown to postpone clinical manifestations, it has been proposed that treatment should begin much earlier in patients - before tissue damage becomes irreversible[9]. Yet such a course of action will significantly drive up costs even more as decades of treatment are added for each patient. To date there has been no trial of early ERT to test this hypothesis although some data indirectly support the idea of early therapy[10,11]. Canada has a relatively high prevalence of this disease due to a large Nova Scotia kindred with 210 FD patients in Canada currently receiving ERT; the annual cost for this treatment approaches $49M[12]. This is a significant cost for a non-curative treatment. Lastly, one can expect the cost of FD treatment to increase further as more patients are diagnosed with this condition.

2.2. Recombinant LV-based Gene Therapy Clinical Trials

Since the first approved human gene therapy trial in 1989 by Rosenberg et al.[13], over 1597 clinical trials have been completed, are ongoing, or have been approved, using over 100 genes[14]. The first LV-based clinical trial, “A Phase I Open-Label Clinical Trial of the Safety and Tolerability of Single Escalating Does of Autologous CD4 T Cells Transduced with VRX496 in Human Immunodeficiency Virus (HIV) Positive Subjects”, was approved in 2001[14,15,16]. The actual phase I trial that started in 2003 was completed in 2006 with five subjects enrolled. This trial involved the first-ever use of a LV vector in humans. It evaluated the safety of a conditionally replicating HIV-derived LV vector expressing an antisense gene against the HIV envelope. The results from this clinical study demonstrated the high efficiency of

gene transfer in vitro at the clinical scale. Sustained gene transfer occurred with no evidence of insertional mutagenesis after 21-36 months observation[15,16].

To date, LV vectors have been approved for use in at least 158 clinical trials, comprising 6.4% of all gene therapy protocols[14]. Clinical successes in the context of inherited single gene defects and hematopoietic cell targets have buoyed enthusiasm for this delivery schema and are informative here: Cartier et al.[17] demonstrated that autologous $CD34^+$ selected hematopoietic cells could be genetically modified *ex vivo* and re-infused into two adrenoleukodystrophy (ALD) patients following myeloablative treatment. The infused hematopoietic cells persisted in a polyclonal nature and expressed the therapeutic ALD protein; this led to a halt in the progression of cerebral demyelination in the patients. Even more recently, Naldini's group published two landmark papers in the field. In the first, they demonstrated that recombinant LV-mediated gene transfer into autologous hematopoietic stem/progenitor cells (HSPC) followed by infusion into conditioned metachromatic leukodystrophy patients (MLD; another LSD) led to high arylsulfatase expression throughout hematopoietic lineages and in the cerebrospinal fluid of these gene therapy recipients for extended periods of time[18]. They went on to show that there was no evidence of aberrant clonal behaviour in the transduced cells and that the MLD did not progress in the three patients 7-21 months beyond the predicted age of onset. To date they have treated 22 patients with this disorder. Lastly, this meta-group showed that such methods and successful outcomes were also portable to another disorder: Wiskott-Aldrich syndrome wherein LV-mediated gene transfer into $CD34^+$-selected HSPC followed by reinfusion led to stable improvement in platelet counts, immune function, and clinical scores[19].

2.3. Pre-Clinical Studies of LV-based Gene Therapy for Fabry Disease

The Medin lab has been instrumental in the demonstration of feasibility of gene therapy for FD and in the development of LV-based gene therapy for this disorder[20,21]. Their primary focus has been targeting cells of the hematopoietic system to effect therapy for FD using integrating viral vectors[22,23,24,25,26,27,28,29]. This approach exploits two facts: that one of the major sources of systemic Gb_3 may be from the breakdown of red blood cells; and that genetically-modified hematopoietic cells can circulate and allow for systemic metabolic cooperativity (or cross-correction) effects. Here, many over-expressed lysosomal hydrolases (including α-gal A) are secreted by modified cells and can be taken up into uncorrected bystander cells via mannose-6-phosphate receptors. In this way, even a low percentage of transduced cells can effect systemic therapy. Recently, the focus has shifted to using recombinant LV vectors due to their ease of application in this context and to their enhanced safety features compared to onco-retroviral delivery schemas. The Medin lab has tested such gene therapy outcomes *in vitro* and *in vivo* in normal and Fabry mice. In the past few years, their primary goal has been focused specifically toward improving gene transfer efficiency and safety[26], along with accumulating key pre-clinical data in existing and newly generated models[24] for eventual clinical trials.

Recently, the Medin lab also completed a pre-clinical safety and efficacy study of gene therapy for Farber disease (another LSD) in three enzymatically-normal non-human primates (NHP, macaques) using a recombinant LV vector that employs the

same backbone as the vector in this current protocol[30]. Autologous, mobilized peripheral blood cells transduced with a LV engineering expression of acid ceramidase (ACDase) were infused into fully myeloablated recipient NHP and tracked for over one year. ACDase specific activity, ceramide levels, vector persistence/integration, and a wide range of safety parameters were assessed. No hematological, biochemical, radiological, or pathological abnormalities were observed in any recipient. Hematological recovery occurred by approximately 3 weeks post-transplantation. Vector persistence, but no clonal proliferation of PB or bone marrow (BM) cells, was observed in all animals throughout the study, thus confirming the safety of this approach. Importantly, ACDase-specific activity was detected above normal background levels in PB and BM cells analyzed post-transplantation and in spleens and livers at sacrifice, indicating the therapeutic feasibility of this approach. In addition, ceramide levels in PB cells and in spleen and liver tissues decreased.

2.4. Recombinant Lentiviral Vector Design

Our group has constructed a novel LV vector that engineers expression of the human α-gal A cDNA. The α-gal A is codon-optimized for enhanced expression in human cells by maximizing corresponding codon usage for existing human transfer RNA (tRNA) pools.

2.5. Study Rationale

ERT has been the main treatment modality for FD for over a decade. This approach has been moderately effective with reduction of Gb_3 in plasma, urine, and tissue as well as reduction of other GSL such as lyso-Gb_3 and related analogues in plasma and urine. In addition, clinical stabilization of both renal and cardiac disease occurs in many patients[12,31,32]. There is also subjective improvement with reduction in neuropathic pain and gastrointestinal symptoms and increased sweating. Unfortunately, ERT is not a cure and many patients continue to have symptoms with progressive disease despite treatment[33]. ERT is also extremely costly and puts a high lifelong cost burden for FD patients and the Canadian health care system. Patients receiving ERT need bi-weekly infusions of the product, which carries issues of inconvenience, infusion-associated reactions (IAR), anti-agalsidase antibodies, and venous access. Recent production issues with global shortages of ERT and changes in drug licensing status in Canada highlight the fragility of the current treatment infrastructure for FD.

As an alternative, gene therapy involving a variety of gene delivery systems has been pursued pre-clinically for the amelioration of FD. Especially attractive here are delivery systems that may provide long-term correction with only a single application. FD is a compelling disorder for gene therapy, as target cells for modification are readily accessible and relatively low levels of enzyme correction may suffice to reduce substrate storage. Importantly (as mentioned above), metabolic cooperativity or 'cross-correction' effects are also manifested in FD, wherein corrected cells allow secretion of α-gal A to correct unmodified bystander cells. We have performed many pre-clinical studies using integrating delivery vectors in the context of FD[18-27] and can target hematopoietic stem cells to enable their progeny to renew and

differentiate into all blood cells, thereby allowing corrective enzyme to be distributed systemically over time.

LV vector-based gene therapy offers advantages over conventional onco-retroviral (aka gamma-retroviral) gene delivery systems and has become a popular choice for clinical gene transfer *ex vivo* approaches[34],[35]. LV stably integrates transgenes into somatic host genomic DNA leading to stable protein expression that is passed down to daughter cells. Current technology allows for up to 20kb of transgene DNA transfer to host cells. This occurs without transfer of viral specific genes, unlike other vector systems. LVs are considered safe and have the ability to transduce both dividing and non-dividing cells with remarkably high efficiency. Expression is long-lasting even after stem cell differentiation[36,37,38]. Additional benefits include resistance to transcriptional silencing, and no preference for transcriptional start sites, including DNase 1 hypersensitivity sites and CpG islands[39]. Finally, there is a lack of interference from pre-existing viral immunity and a lack of precedence linking LV vectors with tumour formation[40,41]. Most LV vectors used currently (including the vector used in this trial) have a 3′ long-terminal repeat (LTR) self-inactivating (SIN) design. Here the 3’ LTR is inactivated, thus when it is converted to the 5′ LTR upon reverse transcription following cell infection and nuclear import, the subsequent 5′ LTR promoter of the integrated provirus will be inactivated. Current LVs are also packaged using multiple plasmid transfections, which further reduce the chance of formation of replication-competent LV (RCL).

Up to 6 adult male patients with confirmed FD currently receiving ERT will be enrolled into this study. Heterozygote females are excluded, as the majority will have a mild Fabry phenotype that does not require any specific therapy. Similarly, males with variant FD are excluded as they may have milder disease of later onset and the benefit of ERT is undefined in those cohorts.

Our experience using Melphalan monotherapy as a conditioning regimen has been in patients with plasma cell neoplasms. This 20 year experience includes patients up to the age of 75 with multiple myeloma, primary amyloidosis and rarely POEMS syndrome. Collectively, the risk of mortality is well below 1% in this cancer population who often have cumulative hematological toxicity, abnormal renal function, peripheral neuropathy and bone lesions. Our experience (>250 myeloma transplants/year at PMH) has taught us that although age is a factor, medical fitness and objective evaluation of organ function is an effective strategy to predicting regimen related toxicity and safety. To this end in this Fabry pilot study, we have developed a comprehensive list of exclusion criteria that focus on adequate organ function. Moreover, we are using only 50% of the dose Melphalan (100mg /m2). The FACT study team and bone marrow transplant physicians have discussed this issue in considerable detail and unanimously agree allowing patients meeting all inclusion and exclusion with age 18-50 is acceptable and safe.

3. TRIAL DESIGN

This is a multi-centre, non-randomized, open-label, prospective pilot study of a novel stem cell gene therapy protocol in up to 6 adult male patients with confirmed FD currently receiving ERT. Up to three accredited Regional Stem Cell Transplant Centres (RSCTC) in Canada will be used in this study with a local Qualified Investigator (QI). The overall goal of this pilot project is to establish the safety of stem cell gene therapy for male patients with FD.

The study will be conducted in four phases: Phase 1 - Screening, Phase 2 - Pre-Mobilization, Mobilization and approval of CoA phase, Phase 3 -Treatment phase and Phase 4 - Post-Treatment Follow Up phase. The Screening phase occurs within 90 days prior to the Pre-Mobilization Phase. Eligible patients (based on screening assessments in Phase 1) will be registered into the trial prior to starting Phase 2 (the phase to start research procedures to prepare for final product). Phase 2 consists of 6 stages: Pre-Mobilization, Mobilization, Leukapheresis, CD34+ selection, Transduction & Cryopreservation and Certificate of Analysis Approved by Health Canada. Patient enrollment into the study will occur after day -2 assessments of Treatment Phase have been performed and patient is deemed fully eligible to receive protocol treatment (ie: CoA approved by Health Canada and patient is suitable for transplant per day -2 assessments). Phase 3 Treatment phase consists of Transplant stage. After patients complete the Treatment phase, they will enter the Post-Treatment Follow up phase and be followed at a regional FD cancer center for 5 years post-transplant. Refer to Figure 1 and Study Calendar in section 5.

Figure 1: Figure 1 shows the 4 different study phases along with the location of each phase (and stages) and the study timeline. For more details, refer to Study Calendar in section 5.

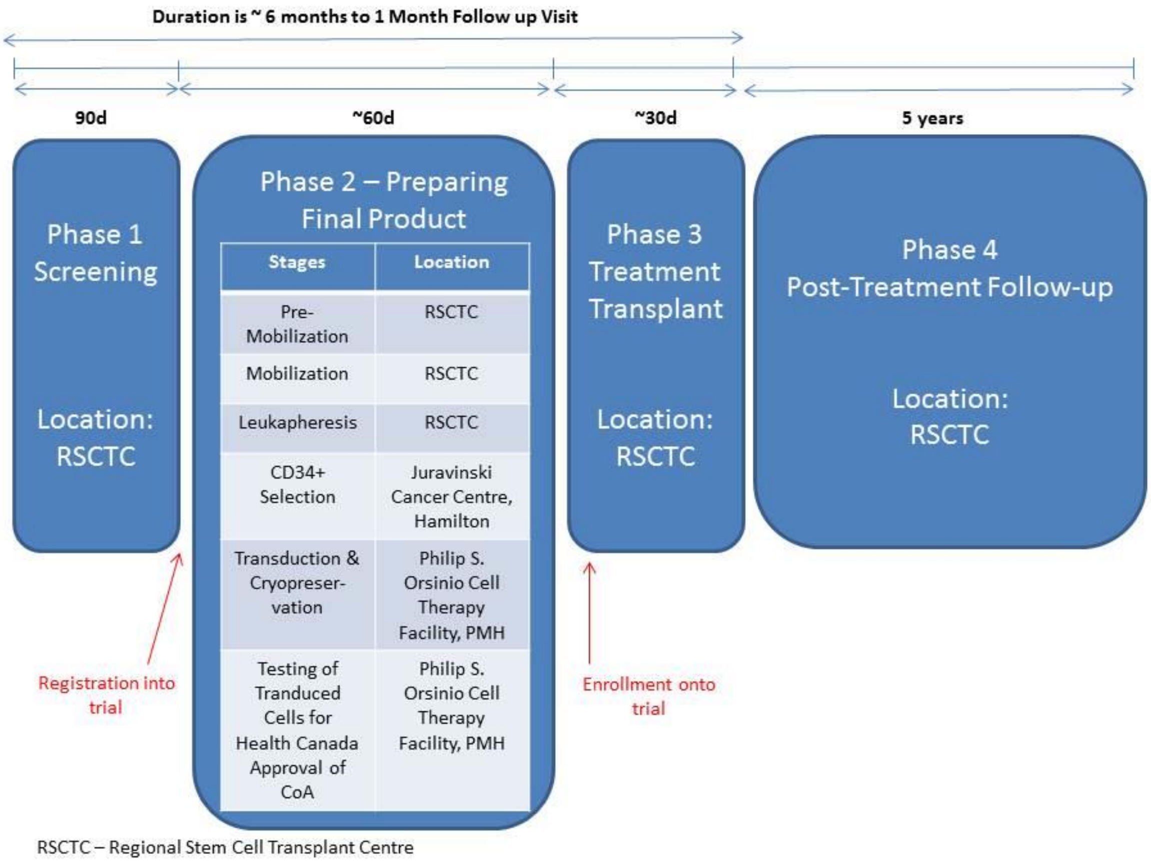

4. PATIENT POPULATION

This trial will be conducted in compliance with the protocol, GCP and the applicable regulatory requirement(s).

Eligibility status must be confirmed by the co-Investigator or designate prior to enrolment. No exceptions will be made to the eligibility criteria. Questions related to eligibility requirements and/or specific criteria must be discussed with the study's Medical Monitor (on the cover page) prior to patient registration.

4.1. Inclusion Criteria

For inclusion in this study, patients must fulfill ***all*** of the following criteria:

1. Male patients 18–50 years of age at the time of enrollment
2. Diagnosis of FD as defined by very low (<7.0% of mean) or absent α-gal A activity in plasma or leukocytes as measured in Dr. Rupar's laboratory at London Health Sciences Centre (screening sample must be sent to Dr. Rupar's lab to determine eligibility for this inclusion criterion)
3. Classic FD Type I phenotype with GLA genotyping

4. Patients on ERT for minimum of 6 months prior to enrollment
5. Eastern Cooperative Oncology Group (ECOG) Performance status of 0 or 1
6. Adequate organ function within 90 days prior to Pre-Mobilization Phase:

   -liver: serum bilirubin < 1.5× upper limit of normal, aspartate aminotransferase/ alanine aminotransferase (AST/ALT) < 3× upper limit of normal;
   -renal: Adequate renal function (estimated GFR (eGFR) by CKD-epi formula[1] >45 ml/min/1.73m^2
   -pulmonary: diffusing capacity of the lung for carbon monoxide (DLCO), forced expiratory volume in 1 second (FEV1), forced vital capacity (FVC) >50% predicted value (corrected for hemoglobin);
   -heart: left ventricular ejection fraction (LVEF) at rest >45%, fractional shortening >0.25%

7. Willing and capable of signing and giving written informed consent in accordance with Research Ethics Board (REB) requirements
8. Willing to comply with all procedures outlined in the study protocol, cooperative with the protocol schedule, able to return for safety evaluation, or otherwise likely to complete the study
9. Willing to abstain from sexual activity or willing to use double-barrier method during sexual intercourse from day of Melphalan administration until 12 months follow-up post-transplant. Sexually active male patients will use condoms and in addition, should ask their female partners with child-bearing potential [defined as a sexually mature woman who has not undergone hysterectomy or who has not been naturally postmenopausal for at least 24 consecutive months (i.e. who has had menses any time in the preceding 24 consecutive months)] to use oral, implantable or injectable contraceptives, contraceptive patch, intrauterine device, diaphragm with spermicidal gel when having sexual intercourse. Refer to Section 6.9.
10. Willing to not donate sperm after receiving Melphalan. Sperm banking will be recommended to any patient who would like to father children in the future.

4.2. Exclusion Criteria

Patients meeting ***any of the following exclusion criteria are NOT eligible for this study:***

1. Males with variant Fabry Disease.
2. Female gender
3. Use of immunosuppressive agents or any anticoagulant (warfarin, dabigatran, or other oral anticoagulant, and heparin); antiplatelet agents allowed but may need to be withheld in the presence of bleeding or platelet counts <50x10^9/L
4. Ongoing ERT-related IAR of moderate-to-severe intensity

5. Blood test positive for HBV, HCV, HIV, HTLV-1/2 or VDRL (Transmissible Disease testing will be done in Pre-Mobilization Phase 2 – see section 5.1 for full panel of TD tests. Patients will only be excluded from the study if positive for the TD tests listed here in this exclusion).
6. Uncontrolled bacterial, viral, or fungal infections
7. Prior malignancies except resected basal cell carcinoma
8. Chronic Kidney Disease (CKD) stage >3A (eGFR by CKD-epi <45 mL/min/1.73m^2)
9. History of heart failure or LVEF <45% or moderate to severe diastolic dysfunction by standard criteria
10. Arrhythmia: bundle branch block, heart block degree II or III, atrial fibrillation, supraventricular tachycardia, ventricular tachycardia, ventricular fibrillation, cardiac arrest, pacemaker, implantable cardiac defibrillator
11. Coronary artery disease with angina, prior myocardial infarction, percutaneous transluminal coronary angioplasty with or without stent, coronary artery bypass graft surgery, moderate to severe valvular heart disease, valve replacement surgery
12. Prior stroke or transient ischemic attack (TIA); or stroke on prior brain imaging
13. Uncontrolled hypertension ≥150/90
14. Diabetes mellitus
15. Advanced liver disease, liver failure, cirrhosis
16. Immune deficiency state
17. Moderate-to-severe chronic obstructive pulmonary disease (COPD)
18. Any hematological condition with white blood cells (WBC) <3.0 x10^9/L, platelet count <100 x10^9/L, and/or hemoglobin <100 g/L
19. Prior BMT or organ transplant
20. Any condition that would preclude use of Melphalan
21. Use of a drug with cytotoxic or immunosuppressive effect within 60 days of trial entry
22. Uncontrolled psychiatric disorder
23. Active chronic infection
24. Prior tuberculosis
25. Any other serious concurrent disease in the investigator’s opinion, that could affect study endpoints
26. Cognitive impairment that would prevent informed consent
27. Use of an investigational drug within 30 days of SCT

4.3. Patient Registration and Enrollment

All patients will be screened by one of the principal investigators or sub-investigators prior to entry into this study. An explanation of the study and discussion of the expected side effects and full disclosure of the “informed consent” document will take place. Consented patients and patients meeting eligibility criteria from the screening phase (Phase 1) will be registered into the study before starting Pre-Mobilization phase.

Registration will be done through Ozmosis Research Inc. Sites will assign each patient with a patient ID number which should be used on all documentation and correspondence.

Prior to registering a patient, each institution must have submitted all necessary regulatory documentation to Ozmosis Research Inc. Access to the eCRFs will only be granted once this has been received. All sites should call Ozmosis Research Inc. Clinical Trials Manager (CTM) at the number listed on the front page to verify study availability.

No patient can start Phase 2 Pre-Mobilization phase of this study until eligibility from screening phase has been confirmed and the Patient Registration and Enrollment Fax has been submitted to Ozmosis Research Inc. Eligibility criteria from the screening phase must be met at the time of registration. There will be no exceptions. Any questions should be addressed with Ozmosis Research Inc. prior to registration.

The Patient Registration and Enrollment Fax must be completed, and signed by the investigator prior to registration and enrollment. There are 8 sections to the Patient Registration and Enrollment Fax. Fax or email to Ozmosis Research at 416-598-4382 or ozmclinical@ozmosisresearch.ca.

1) SCREENING: This section is completed by the site and should be faxed or emailed to Ozmosis at the time of screening.

2) REGISTRATION: This section is completed by the site at the time of registration. The site will fax or email the signed and completed Patient Registration and Enrollment Fax to Ozmosis Research Inc. Only after this has been sent to Ozmosis can the patient start Pre-Mobilization phase. **It is the responsibility of the investigator in charge to satisfy him or herself that the patient is indeed eligible before requesting registration.**

   All eligible patients registered into the study will be entered into a patient registration log at Ozmosis Research Inc.

3) CONFIRMATION OF REGISTRATION (TO BE COMPLETED BY OZMOSIS): Confirmation of Registration must be received by site from Ozmosis prior to patient initiating Pre-Mobilization phase.

4) PRE-MOBILIZATION PHASE: This section is completed by the site at the completion of Pre-Mobilization phase on D28 and faxed or emailed to Ozmosis Research Inc.

5) MOBILIZATION: This section is completed by the site and faxed or emailed to Ozmosis Research Inc. upon shipment of the apheresis product to Juravinski Hospital.

6) CERTIFICATE OF ANALYSIS APPROVAL BY HEALTH CANADA (TO BE COMPLETED BY OZMOSIS): Certificate of Analysis approval by Health Canada must be received by site from Ozmosis prior to patient initiating transplant phase.

7) ENROLLMENT: This section is completed by the site and faxed or emailed to Ozmosis Research Inc. after it has been determined that the patient is fully eligible to take part in the study (ie: CoA approved by Health Canada and D-2 assessments have completed and investigator deemed patient suitable for transplant). This should be done prior to administration of Melphalan on D-1 and study treatment on D0 to the patient. Fax or email to Ozmosis either on D-2 after investigator deemed patient suitable for transplant or on D-1 before Melphalan administration).

8) CONFIRMATION OF ENROLLMENT (TO BE COMPLETED BY OZMOSIS): Confirmation of ENROLLMENT must be received by site from Ozmosis prior to patient receiving Melphalan and protocol treatment.

5. STUDY CALENDAR

Table 1: Study Calendar for Phases 1, 2 and 3: Screening, Preparing Final Product and Treatment Phases

Assessments	Phase 1		Phase 2												Phase 3									Exit Visit (For patients who go off study prior to receiving transplant)[24, 26]
	Screening Phase (w/in 90d prior to Pre-Mobilization)	Registration	Pre-Mobilization Phase (28 days lead-in prior to Mobilization Phase) D1 to 28			Mobilization D1 to 6 or 7							CoA Ap-proved by HC	Treatment Phase: Transplant[24] D-2 to +12 (Enrollment after d-2, before day-1)								D13-27[19] (+/-3 day window[22])	
			1	14	28	1	2	3	4	5	6	7 (optional)	Pass/ Fail	-2[18]	-1	0	1	3	6	9	12		
History of FD[1]	X																						
Vital Signs[2]	X		X[2]	X[2]	X[2]	X				X[2]	X[2]	X[2]		Daily[2]								X[19]	X[24,26]
Physical Exam	X					X								Daily								X[19]	X[24,26]
Weight & ECOG	X					X								X[18]							X	X[19]	X[26]
CBC, differential[3]	X					X				X	X	X		Daily								X[19]	X[26]
Electrolytes[4], urea/BUN, creatinine	X					X				X				Daily								X[19]	X[26]
Serum Chemistry[5]	X					X				X	X	X		X[18]		X		X	X	X	X	X[19]	X[26]
INR, PTT	X					X								X[18]								X[19]	
Serum Troponin, CK	X													X[18]								X[19]	
24hr urine creatinine, protein	X													X[18]								X[19]	
Albumin-to- creatinine ratio																					X		
Urinalysis[6]	X													X[18]								X[19]	
24-hr Holter Monitoring	X													X[28]									
ECG	X													X[18]									
ECHO or MUGA[28]	X[28]													X[28]									
Brain MRI (optional)[20]	X[20]																						
Cardiac MRI (optional)[20]	X[20]													X[20]									
Pulmonary Function Tests[7]	X																						
CXR (or CT chest)														X									

Assessments	Phase 1		Phase 2											Phase 3									Exit Visit (For patients who go off study prior to receiving transplant)[24, 26]
	Screening Phase (w/in 90d prior to Pre-Mobilization)	Registration	Pre-Mobilization Phase (28 days lead-in prior to Mobilization Phase) D1 to 28			Mobilization D1 to 6 or 7							CoA Ap-proved by HC	Treatment Phase									
														Transplant[24] D-2 to +12 (Enrollment after d-2, before day-1)								D13-27[19] (+/-3 day window[22])	
			1	14	28	1	2	3	4	5	6	7 (optional)	Pass/ Fail	-2[18]	-1	0	1	3	6	9	12		
Blood for T/B cell count[21]	X (optional)													X							X	X[19]	
Group and screen						X								X								X[19]	
TD testing[8]				X[8]																			
ERT administration[9]	X[9]		X[9]	X[9]	X[9]																		
Neupogen® (filgrastim) Injection[10]						X	X	X	X	X	X	X (optional)							[10]Day 5 until ANC >1.5 × 10^9/L				
Plerixafor (optional)[23]										X[23]													
Harvest stem cells[27]								X[27]	X[27]	X[27]	X[27]	X[27]											
Pre-absolute CD34 count/µL										X	X	X											
Flow Cytometry[11]										X	X	X											
Melphalan Administration[12]															X								
**Infusion of transduced stem cells[13]**																**X**							
qPCR & Plasma α Gal A activity[14, 15]	X[14]		X	X	X									Daily [25]								X[19]	
α Gal A activity in leukocytes[14, 15]	X[14]		X	X	X											X			X		X	X[19]	
Bone marrow aspirate[15]														X									
Plasma Gb_3 & isoforms & analogues and lyso-Gb3 and related analogues[14, 15]	X		X	X	X									Daily[25]								X[19]	
Urine lyso-Gb3 and related analogues[14,15]	X		X	X	X									Daily[25]								X[19]	
Anti-agalsidase	X		X	X	X																		

**Assessments**	**Phase 1**	**Registration**	**Phase 2**											**Phase 3**									**Exit Visit (For patients who go off study prior to receiving transplant)[24, 26]**
	**Screening Phase (w/in 90d prior to Pre-Mobilization)**		**Pre-Mobilization Phase (28 days lead-in prior to Mobilization Phase) D1 to 28**			**Mobilization D1 to 6 or 7**							**CoA Ap-proved by HC**	**Treatment Phase**									
														**Transplant[24] D-2 to +12** (Enrollment after d-2, before day-1)								**D13-27[19] (+/-3 day window[22])**	
			**1**	**14**	**28**	**1**	**2**	**3**	**4**	**5**	**6**	**7 (optional)**	**Pass/ Fail**	**-2[18]**	**-1**	**0**	**1**	**3**	**6**	**9**	**12**		
antibodies assays[14, 15]																							
Urine Gb_3 & Isoforms & analogues[14, 15]	X		X	X	X									Daily[25]								X[19]	
Optional Correlatives: Urine metabolite assays[16]	Leftover urine from mandatory urine lyso-Gb3 and Gb3 samples will be used for this optional urine study																						
Concomitant Medications	X				X	Continuous																	
Baseline Symptoms	X				X																		
AEs[17]						Continuous																	

Table 2: Study Calendar for Phase 4: Post-Treatment Follow up Phase (Year 1-5)

**Phase 4: # Months Post-Treatment**	**Year 1 (+/-7days window except 1mth FU which has a +/-3days window)**				**Year 2 (+/-14days window)**		**Year 3 (+/-14days window)**	**Year 4 (+/-14days window)**	**Year 5 (+/-14days window)**
	**1 mth**	**3mth**	**6mth**	**12mth**	**18mth**	**24mth**	**Annually**	**Annually**	**Annually**
Vital Signs	X	X	X	X	X	X	X	X	X
PE incl. weight & ECOG	X	X	X	X	X	X	X	X	X
CBC, differential[30]	X	X	X	X	X	X	X	X	X
Blood for T/B cell count[21]	X	X	X	X					
Electrolytes, urea/BUN, creatinine	X	X	X	X	X	X	X	X	X
Serum Chemistry	X	X	X	X	X	X	X	X	X
24hr urine creatinine, protein	X	X	X	X	X	X	X	X	X
Bone marrow aspirate[29, 30]	X[29]								
qPCR & Plasma α Gal A activity[15, 30]	X	X	X	X	X	X	X	X	X
α Gal A activity in leukocytes[15, 30]	X	X	X	X	X	X	X	X	X
Plasma Gb_3 and isoforms and analogues[15, 30]	X	X	X	X	X	X	X	X	X
Plasma lyso-Gb3 and related analogues[15, 30]	X	X	X	X	X	X	X	X	X
Urine lyso-Gb3 and related analogues[15, 30]	X	X	X	X	X	X	X	X	X
Anti-agalsidase antibodies assays[30]	X	X	X	X	X	X	X	X	X
Urine Gb_3 and isoforms and analogues[15, 30]	X	X	X	X	X	X	X	X	X
Urinalysis	X	X	X	X	X	X	X	X	X
Optional Correlatives: Urine metabolite assays	Leftover urine from mandatory urine lyso-Gb3 and Gb3 samples will be used for this optional urine study								
CXR (or CT chest if CT done at screening)			X						
ECG		X	X	X	X	X	X	X	X
24-hr Holter Monitoring			X	X		X	X	X	X
ECHO or MUGA			X	X		X	X	X	X
Brain MRI			X	X					
Cardiac MRI			X	X		X	X	X	X
Pulmonary Function Tests			X	X					
Concomitant Medication	X	X	X	X	X	X	X	X	X
Adverse Events	X	X	X	X	X	X	X	X	X

Table 3: Study Calendar for Patients Who Meet the Criteria* to Stop ERT Administration After 6 Months Post-Transplant

**If ERT is stopped any time *after* 6 months:**	**For 1st Year After Stopping ERT[31]**	**For 2nd Year after stopping ERT[31]**	**For 3rd year after stopping ERT[31]**	**For 4th Year after stopping ERT[31]**	**For 5th Year after stopping ERT[31]**
**If ERT is stopped at 6 months:**	**6 months – 18 months post-transplant (+/-7days window)**	**Year 2 (+/-14days window)**	**Year 3 (+/-14days window)**	**Year 4 (+/-14days window)**	**Year 5 (+/-14days window)**
**Assessments**	**Every 3 Months**	**Bi-annually (every 6 months)**	**Bi-annually (every 6 months)**	**B-annually (every 6 months)**	**B-annually (every 6 months)**
Vital Signs	X	X	X	X	X
PE incl. weight & ECOG	X	X	X	X	X
CBC, differential[30]	X	X	X	X	X
Blood for T/B cell count[21]	X				
Electrolytes, urea/BUN, creatinine	X	X	X	X	X
Serum Chemistry	X	X	X	X	X
24hr urine creatinine, protein	X	X	X	X	X
qPCR & Plasma α Gal A activity[15, 30]	X	X	X	X	X
α Gal A activity in leukocytes[15, 30]	X	X	X	X	X
Plasma Gb_3 and isoforms and analogues[15, 30]	X	X	X	X	X
Plasma lyso-Gb3 and related analogues[15, 30]	X	X	X	X	X
Urine lyso-Gb3 and related analogues[15, 30]	X	X	X	X	X
Anti-agalsidase antibodies assays[30]	X	X	X	X	X
Urine Gb_3 and isoforms and analogues[15, 30]	X	X	X	X	X
Urinalysis	X	X	X	X	X
Optional Correlatives: Urine metabolite assays	Leftover urine from mandatory urine lyso-Gb3 and Gb3 samples will be used for this optional urine study				
CXR (or CT chest if CT done at screening)	X				
ECG	X	X	X	X	X
24-hr Holter Monitoring	X	X	X	X	X
ECHO or MUGA	X	X	X	X	X
Brain MRI	X				
Cardiac MRI	X	X	X	X	X
Pulmonary Function Tests	X				
Concomitant Medication	X	X	X	X	X
Adverse Events	X	X	X	X	X

*Patients may stop ERT administration 6 months post-transplant if the following criteria are met:
1) The patient continues to make his own alpha gal-A enzyme at 6 month post-transplant as determined in the study reference laboratory at a level deemed acceptable by the investigator.
2) The patient is deemed medically stable by their study physician
3) The patient provides consent for withdrawal of ERT following discussion with their study physician, and this has been documentated by the physician and/or nurse that it was discussed with the patient and they are in agreement.
4) The CTSC unanimously agree and provide written approval that ERT may be stopped for the patient after reviewing all relevant data and literature, and evaluating the risks vs. benefits to the patient

All decisions made will be documented and filed along with their rationale. Close monitoring of these patients will be performed as per Table 3 above. Should any of these patients show any safety concerns after stopping ERT, ERT may re-start as per investigator's discretion. Refer to Section 6.4.4.2

5.1. Footnotes

1. History: includes history of FD, prior therapy, past medical and surgical history with outcomes, medications, historical α-gal A, GLA mutational analysis, concurrent medical conditions
2. Vital signs include pulse rate, blood pressure, temperature and respiratory rate. Vital signs will be taken as indicated in the Study Calendar. During Pre-Mobilization phase, vital signs will be done prior to, during and after ERT administration. During the Treatment Mobilization phase, vital signs will be done on day 1, and for days 5 to 7, vital signs will be done prior to collection of the stem cells on each of these days (day 7 is optional). During the Treatment Transplant phase, vital signs will be done daily. On day 0, vital signs will be done prior to infusion and post-infusion at 30 min, 1hr, 1.5hr and 2.0hr timepoints. A window of +/- 10 min is allowed for timepoints on day 0. Additional vital signs may be done as per institutional SOPs or as per discretion of the treating physician.
3. CBC with differential includes: hematocrit, hemoglobin, platelet count, WBC (total and differential), RBC & ANC.
4. Electrolytes include: sodium, potassium, chloride and bicarbonate.
5. Serum Chemistry includes: AST, ALT, ALP, LDH, bilirubin, calcium, albumin, phosphate, magnesium, uric acid, amylase, glucose, GGT.
6. Urinalysis includes: blood, glucose, protein, specific gravity & microscopic exam (if abnormal).
7. Pulmonary Function Tests include: 1) Forced expiratory volume in one second - FEV1 (L/sec), 2) FEV1 , % of predicted, 3) Forced Vital Capacity, 4) Forced Vital Capacity, % predicted, 5) FEV1/FVC ratio, 6) FEV1/FVC ratio, % predicted, 6) Total lung capacity - TLC (L), 7) TLC, % of predicted
8. Transmissible disease (TD) testing include: Health Canada approved serological analysis for HIV Ag/Ab Screen, HBs Ag screen, Anti-HBcore, Anti-HCV screen, Syphillis test (VDRL), Cytomegalovirus (CMV) IgG Ab, CMV IgM Ab, HTLV ½ Ab screen, HSV-1 IgG Ab, and as applicable VSV IgG Ab and West Nile Virus (PCR). This testing is required as per FACT/Health Canada and must be done within 30 days of apheresis collection day 2 or 3 (day 3 is optional). TD testing can be done at any time during the Pre-Mobilization phase as long as these tests are done within 30 days of apheresis collection day 2 or 3 (day 3 is optional). Patients who are positive for HBV, HCV, HIV, HTLV-1/2 or VDRL will be removed from the study (Refer to section 4.2).
9. ERT administration (agalsidase beta or agalsidase alfa) will be given in the Pre-Mobilization phase on the days indicated in the study calendar. ERT may stop on day 28 of Pre-Mobilization Phase (at least 30 days prior to ASCT on day 0) and will resume administration at 30 days post-transplant in Phase 4 of the study. Refer to section 6.4.2.1. Flexibility around the timing of ERT administration will be allowed as per treating physician.
10. Neupogen® (filgrastim) will be given on the days indicated in the study calendar subcutaneously or as per physician's discretion. Neupogen may be self-administered by the patient after appropriate training for self-administration is provided by qualified staff. During Mobilization phase, patients will be given at a dose of 16µg/kg recipient weight (rounded off to nearest vial size – vial sizes are 300ug and

480ug) of filgrastim. During transplant phase, dose is 5µg/kg and may start as per physician's discretion until neutrophil count is >1.5x10^9/L in transplant phase, then filgrastim can be stopped.

11. Flow cytometry will be used for end of collection CD34 enumeration. A minimum of 10.0 × 10^6 $CD34^+$ cells/kg weight will be collected or patient is not eligible for the study. Plerixafor may be administered if min 7.5x10^6 CD34+ cells/kg weight cannot be collected on first day of collection in Mobilization phase. Refer to section 6.4.2.3.
12. Melphalan will be administered I.V. at 100 mg/m^2 and will be given in a single dose as per institutional procedures. Study infusion must not be administered until at least 24hrs after completion of the Melphalan administration.
13. The infusion of transduced autologous stem cells will occur on day 0 within the Treatment Transplant phase (refer to section 6.4.3). Study infusion must not be administered until at least 24hrs after completion of the Melphalan administration.
14. In the Screening phase, the peripheral blood taken for plasma α Gal A activity and α Gal A activity in leukocytes must be collected no sooner than 5 days after ERT infusion, processed and sent as per lab manual as soon as possible to Dr. Rupar's lab to determine eligibility of inclusion criteria #2. In the Pre-Mobilization phase, the peripheral blood and urine taken for these tests (plasma α Gal A activity, α Gal A activity in leukocytes, CD19+ lymphocytes, plasma Gb_3, Plasma lyso-Gb_3 and related analogues, urine lyso-Gb3 and related analogues, anti-agalsidase antibodies assays and urine Gb^3) must be drawn **prior** to ERT administration on the same day for trough levels. Urine samples for Gb3 and isoforms and analogues and lyso-Gb3 should be taken in the morning.
15. Peripheral blood will be collected for qPCR, anti-agalsidase antibodies assays, plasma α-Gal A activity and α-Gal A activity in leukocytes, plasma Gb3, plasma lyso-Gb3 and related analogues. Bone marrow aspirate samples collected will be tested for α-Gal A activity, methyl-cellulose PCR assays and qPCR assay. Urine will be collected for urine Gb3 and urine lyso-Gb3 and related analogues Refer to the lab manual for collection, processing and shipment details.
16. Leftover urine from mandatory urine lyso-Gb3 and Gb3 samples will be used for this optional urine study. No additional urine will be taken for optional correlative studies. Refer to section 9.4.2.1 and lab manual.
17. Adverse Events will be coded as per CTCAE v4.03. For AE and SAE reporting, refer to Section 10.
18. If on day-2, there are significant Fabry related changes in the patient's health status (health jeopardized with proceeding with transplant), a discussion may be warranted with the Clinical Trial Steering Committee to decide on whether or not patient can continue onto the trial. Refer to section 6.1.2.
19. Patients may be managed as outpatient or admitted if deemed medically necessary, if there are ongoing AEs that require close monitoring at the discretion of the treating physician. Assessments will be done as clinically indicated and frequency and duration of monitoring determined at the discretion of the treating physician. If on or after day 21, there is evidence of a failure to engraft (defined as absolute neutrophil count (ANC) < 0.2 × 10^9 cells/L or platelet count <10 x 10^9/L on or after day 21), a discussion will take place between the CTSC members and decide on whether or not to infuse the back-up autograft of non-transduced, non-$CD34^+$ selected cells into the patient. Refer to section 6.4.2.3.
20. Brain and cardiac MRI may be done within 6 months prior to Pre-Mobilization phase, as clinically indicated, upon discretion of the treating physician at Screening. Cardiac MRI must be repeated within 30 days prior to day 0 or date of protocol treatment (transplant).

21. Peripheral blood will be taken for T and B cell count at the timepoints indicated in the study calendar. T and B cell count may include an optional assessment of immune reconstitution pre and post-transplant by routine flow cytometric analysis of peripheral blood lymphocyte subsets. Panel includes: CD3; CD4; CD8; CD19; CD20; CD25; CD56; CD57.
22. During the transplant phase D13-27, a window of +/- 3 days is allowable as per local resources, at the discretion of the investigator.
23. Plerixafor may be administered at 240µg/kg subcutaneously for a maximum of 3 doses, if needed, to a patient where a minimum of 7.5×10^6 CD34+ cells/kg weight cannot be achieved on first day of collection. Plerixafor must not be given to a patient whose WBC level is high – a level at the discretion of the investigator/institution. Refer to section 6.4.2.3.
24. An exit visit will be conducted for patients who receive G-CSF in the Mobilization phase and goes off study prior to receiving the transplant. If a patient goes off study after receiving the transplant, subsequent assessments and follow up will be done as per Study Calendar unless the patient withdraws from any further study follow up.
25. Efforts will be made by each site to collect correlative samples daily (window +/- 72hrs is allowed). Shipments will be done as per Lab Manual.
26. An exit visit will be conducted for patients who receive G-CSF and Melphalan and goes off study prior to receiving the transplant. If this occurs, the back-up, non-selected PBPC maintained at each site during Mobilization phase will be re-infused into the patient. The patient will undergo all assessments as per SOC like any other stem cell transplant recipient. Refer to section 6.7.
27. Stem cells may be harvested on any day (s) between day 3 to day 7 of Mobilization stage, as deemed appropriate by investigator.
28. ECHO or MUGAs done within 6 months of Pre-Mobilization phase day 1 is acceptable. Within 30 days prior to day 0 or protocol treatment (transplant), 24-hr holter monitoring and ECHO or MUGAs must be repeated.
29. Bone marrow aspirate must be taken prior to re-starting ERT.
30. Additional Optional Research Samples after 1 month follow up visit (above what is already required in Table 2 above) - the following additional samples may be taken for research at timepoints determined by the CTSC if patient consents:
    a) Bone marrow aspirate: Year 1 – one additional bone marrow aspirate may be obtained. Years 2-5 – not to exceed more than once a year.
    b) Skin biopsy: not to exceed more than once a year
    c) Blood: not to exceed an additional 350 mL per year (and not more than 160ml during a period of 4 weeks)
    d) Urine: not to exceed an additional 300mL per year

    Efforts will be made to obtain any additional samples only if clinically and scientifically relevant as determined by the CTSC and only if the patient consents to the collection of these samples.
31. Study Calendar to be followed only until 5 years of follow up have been completed.

6. TREATMENT PLAN

6.1. Enrollment Procedures and Safety Review Meeting

Patients will be enrolled across three sites in Canada. After each patient completes the 1 month post-transplant follow-up visit, a safety review meeting will take place with the Clinical Trial Steering Committee (CTSC) (refer to section 6.1.2 and 6.1.3) and Ozmosis Research Inc. to review the safety data (refer to section 6.2). Efforts will be made to have as many CTSC members attend the safety cohort review meeting as possible but at minimum, 2 lead clinical PIs, Medical Monitor and the Project PI should be present. Safety data and minutes will be distributed to all members of the CTSC and any member not present at the CTSC (including if minimum requirements for attendees are not met) will have an opportunity to provide any comments before re-opening the next cohort.

6.1.1. Safety Stopping Rules

Accrual will be on hold and a meeting held with the CTSC and DSMC if the following occurs:

- If there is a death or unacceptable toxicity attributed to be possibly, probably or definitely related to the experimental product
- If there is no evidence of vector copy number in blood or bone marrow or evidence of enzyme activity by day 28 of the Transplant phase
- If there is a protocol treatment-related Serious Adverse Event (any SAEs including failure to engraft deemed by CTSC to be definitely, probably or possibly related to infusion of transduced autologous $CD34^+$ cells), or any significant safety concerns

A decision will be made by the CTSC and DSMC regarding whether the study will be re-opened for the next patient in the study or not. If there is disagreement in the SAE causality assessment or whether or not to re-open accrual between the different parties, the final decision will rest on the DSMC. Ozmosis Research will notify all sites and applicable parties on the decision and whether accrual has re-opened. Only when it is deemed safe can the next patient start the treatment phase of the study.

If a patient withdraws or drops out of study prior to receiving the transplant for any reasons, then another patient can start study right away, provided the patient has signed informed consent and is eligible for the trial. Safety review meetings will review data on patients who have received the transplant.

6.1.2. Clinical Trial Steering Committee (CTSC)

The Clinical Trial Steering Committee consists of all the lead clinical PIs of each participating site, the laboratory investigators, the Medical Monitor and the Project PI. This Steering Committee is responsible for the overall conduct of the trial, including the design, execution, analyses, and reporting. In addition, the Steering Committee is also responsible for the assignment of responsibilities to any other study subcommittees. The

Steering Committee will hold the primary responsibility for publication of the study results (Refer to Section 16.2). This Committee will convene on a regular basis at every Safety Review Meeting by teleconference or face-to-face meetings to address policy issues, to monitor study progress, execution and management, and to review reports from the DSMC and the CRO. A current list of CTSC members is maintained by the Project PI/Sponsor.

6.1.3. Data Safety Monitoring Committee (DSMC)

An independent Data Safety Monitoring Committee has been established by the Clinical Trial Steering Committee. The DSMC will comprise of clinical trial experts in stem cell transplantation, gene therapy, and FD independent of the study. A list of the DSMC members is maintained by the Project PI/Sponsor.

The DSMC will monitor the safety aspects of the trial including review of all SAEs and data during the Safety Review Meeting after the first, third and sixth patient complete 1 month post-transplant follow up visit. Additional meetings may be scheduled as necessary. If there is disagreement within the CTSC in the SAE causality assessment, or whether or not to re-open accrual or other safety issues, the final decision will rest on the DSMC. Please refer to the DSMC Charter.

6.2. Data Required for Safety Review Meeting

Within 3 working days of the first patient completing the 1 month follow-up visit, the following sections of the eCRF and source documentation for laboratory results, must be completed and submitted to the Clinical Trials Specialist/Manager at Ozmosis Research Inc.:

- Study Treatment Infusion
- Adverse Events (including any infections)
- Hematology and Biochemistry Results
- Lab normal ranges page, if applicable

Additional information may also be requested by Ozmosis Research Inc. from the study site. It is imperative that the eCRF pages listed above are completed at Ozmosis Research Inc. within 3 working days after the patient has completed the 1 month follow up visit, as the information will be used to assess safety before the next patient can start treatment phase of the study.

6.3. Cardiology Review

A review of all cardiac related evaluations will be performed for all enrolled patients who received the protocol treatment. This includes but not limited to the holter monitoring evaluations, ECGs, cardiac MRIs, ECHOs or MUGAs performed prior to and in the course of the patient’s trial participation. Each patient’s cardiac history should be very carefully reviewed prior to patient registration into the trial. For any patients being referred to another institution, detailed patient medical history should be forwarded to the participating site administering the protocol treatment at the time of referral. The treating institution will forward such cardiac related evaluations to the cardiologist for review after each enrolled patient completes 6 month follow up post-

transplant visit.

6.4. Study Phases

6.4.1. Phase 1: Screening (within 90 days prior to Pre-Mobilization phase)

Patients who have signed the study informed consent will be screened at the site to determine eligibility. All screening assessments (as per Study Calendar) are to be completed within 90 days prior to the Pre-Mobilization phase of the study. No study activities will occur prior to patients signing consent.

6.4.2. Phase 2: Preparing for Final Product

6.4.2.1. Stage 1: Pre-Mobilization (28 days lead-in prior to Mobilization phase)

Eligible patients will enter a 28-day lead-in period in the Pre-Mobilization phase of study at the site.

Testing during this 28 day lead-in period will include establishment of plasma and leukocyte α-gal A activity, levels of anti-agalsidase antibody, plasma/urine Gb_3 levels, and levels of plasma/urine lyso-Gb_3 and related analogues (refer to protocols provided in the Investigators Brochure/Product Monograph and lab manual). These samples will be collected as indicated in the Study Calendar and **prior to** administration of ERT, to establish baseline levels that will be used to compare to levels post-transplantation.

Transmissible Disease testing will also be conducted during this stage and this must be done as per Health Canada regulations, ***within 30 days*** of apheresis collection day 2 or 3 (day 3 is optional). Refer to study Calendar.

Enzyme Replacement Therapy (ERT)

During this phase, patients will continue to receive their usual ERT alone (agalsidase alfa 0.2 mg/kg or agalsidase beta 1.0 mg/kg given intravenously (I.V.)) on the days indicated in the Study Calendar. ERT will stop on day 28 of Pre-Mobilization phase (at least 30 days prior to day 0 of Transplant phase) and will resume administration at approximately 30 days post-transplant in Phase 4 of the study. If criteria for stopping ERT administration at 6 months post-transplant are met, ERT treatment may be discontinued. Refer to Section 6.4.4.2 .

ERT may be received in the same method as patients have been receiving them locally.

Agalsidase beta (Fabrazyme®, Genzyme, a Sanofi Company, Cambridge, MA USA) is given at a dose of 1.0 mg/kg I.V. every 2 weeks. Agalsidase beta should be administered and stored as per the product label and product monograph. The patient should receive an anti-pyretic agent (acetaminophen 650 mg) orally before each infusion. This may be stopped eventually if there are no IARs. The initial dose should be at 0.5 mg/kg, no faster than 0.01 mg/min. The infusion rate should be gradually increased over subsequent treatments as tolerated up to a maximum rate of 1.0 mg/kg, no faster than 0.25 mg/min over a minimum of 90 minutes. Vital signs are monitored before, during and after infusion. Patients should be observed for evidence of IARs. For further details, refer to the product monograph.

Agalsidase-alfa is currently an investigational product in Canada via REP-081 study sponsored by Shire Inc. but may be marketed in the near future. Patients on agalsidase alfa will be administered according to the Investigator's Brochure until this product becomes marketed, in which case, the Product Monograph will be followed. Those patients who were taking investigational agent agalsidase alfa pre PBSCT will be switched to agalsidase beta 1.0 mg/kg given I.V. every two weeks starting at 30 days post-transplant. Once agalsidase alfa becomes a marketed product, this switch to agalsidase beta will not be required.

6.4.2.2. Stage 2: Mobilization (Days 1 to 6 or 7: Day 7 is Optional. If insufficient number of cells are collected on Days 5 and/or 6, then there may be an additional collection day on day 7)

Peripheral blood $CD34^+$ cells will be mobilized using the G-CSF Neupogen® (filgrastim). Patients will receive 16 µg/kg Neupogen® (rounded off to nearest vial size) subcutaneously (s.c.) each morning for two to four days prior to collection and on each day of collection for a maximum of seven days (refer to study calendar) or as per physician's discretion. The goal is to obtain at total minimum of 10.0×10^6 CD34+ cells per recipient weight kg over 1-3 days (days 5-6 or 7 optional).

6.4.2.3. Stage 3: Leukapheresis (Days 3, 4, 5, 6 or 7)

Patients will undergo leukapheresis using a Spectra Optia® (COBE® Spectra) or other similar approved machine according to the SOP for collection of stem cell products at the Regional Stem Cell Transplant Centre. Collection may be done as early as Mobilization Day 3. A maximum of 3 collections (1 per day) are permitted.

Briefly, leukapheresis will be carried out through central or peripheral venous access. An assessment will be made by the apheresis staff to determine if access through a peripheral vein access is feasible. If it is not, as per standard procedures, a double lumen catheter may be placed in the femoral, subclavian, or internal jugular vein. CBC, and differential and other laboratory investigations according to the Study Calendar will be conducted.

A minimum total of 10.0×10^6 $CD34^+$ cells/kg weight will be collected into sterile, disposable, single-use, leukapheresis bags. If necessary, a second or third leukapheresis product will be collected to obtain the minimum number of cells. **In the event an insufficient number of cells (<10.0×10^6 $CD34^+$ cells/kg weight) are collected, the patient will not be eligible to proceed in the trial and the collected cells will be discarded or donated to research as per the patient's wishes on the informed consent form.** From this, a back-up product containing a minimum of 2.5×10^6 $CD34^+$ cells/kg weight will be processed and cryopreserved at the local transplant center using standard operating procedures and maintained for 10 years. An option to discard or donate the cells to research after 10 years post-transplant will be presented to patients at the time of consent. The remaining PBPC product(s) consisting of a minimum of 7.5×10^6 $CD34^+$ cells/kg weight total will be transported immediately same day to undergo $CD34^+$ selection using a CliniMACs device at the Juravinski Health Sciences Centre in Hamilton the following day. Refer to the Apheresis Product Transport Manual.

Use of Plerixafor

Plerixafor may be administered at 240µg/kg to a patient where a minimum of 7.5×10^6 CD34+ cells/kg weight cannot be achieved on first day of apheresis collection. Plerixafor must not be given to a patient whose WBC level is high – a level at the discretion of the investigator/institution. A maximum of 3 doses is permitted.

Any changes to the minimum number of cells (10×10^6 CD34+ cells/kg weight) required in order to proceed in the trial or other significant issues will be discussed with the CTSC and decisions with rationale documented.

6.4.2.4. Stage 4: CD34+ Selection/Enrichment (at Juravinski Health Sciences Centre in Hamilton)

$CD34^+$ cell enrichment will be performed in a closed system using the CliniMACS® $CD34^+$ Reagent System (Device ID No. 431860; Miltenyi Biotec, Inc) at the Juravinski Health Sciences Centre, Hamilton, Ontario. The CliniMACS® utilizes super-paramagnetic particles composed of iron oxide and dextran conjugated to monoclonal antibodies. These antibodies bind to target cells with the corresponding cell surface antigen (in this case, CD34). After magnetic labelling, the cells are separated using a high-gradient magnetic separation column, which is part of a closed disposable tubing set that is used during the separation procedure. The magnetically labelled cells are retained on the column. Removing the magnetic field elutes the retained cells. Eluted cells are characterized using fluorescence-activated cell sorting (FACS) to determine the purity of the product.

A minimum of 7.5×10^6 $CD34^+$ cells/kg weight will undergo $CD34^+$ selection. All selected cells will be transported at room temperature immediately to the Philip S. Orsino Cell Therapy Facility at Princess Margaret Hospital for LV transduction.

6.4.2.5. Stage 5: Transduction and Cryopreservation (at Orsino Cell Processing Facility in Princess Margaret Hospital)

This protocol is based on personal communications with Biffi and Naldini from Milan and their published protocols[18,19] with modifications based on our own pre-clinical assessments. Briefly, $CD34^+$ cells that have been received from Juravinski Cancer Centre will be placed in stem cell growth media containing cytokines at a concentration of 1×10^6 cells/mL for 24 hours of pre-stimulation. Cells will be transduced a single time with the LV according to the SOP. Cells will be subsequently collected, washed, frozen and kept in vapour phase of liquid nitrogen while testing is completed for Health Canada product release.

LV production batches will be tested for the absence of replication-competent LV by the Good Manufacturing Practice (GMP)-virus producer (Indiana University Vector Production Facility [IUVPF]) according to their SOPs and their Master File with Health Canada. This is done prior to receiving the LV at Princess Margaret Hospital.

Cryopreservation

Transduced $CD34^+$ cells will be promptly cryopreserved in 10% dimethyl sulfoxide by freezing and stored in the vapour phase of liquid nitrogen according to our established SOP. Cryopreserved CD34+ cells will be shipped to the Regional Stem Cell Transplant facility once Health Canada fax-back approval has been obtained.

6.4.2.6. Stage 6: Approval of Certificate of Analysis (CoA) from Health Canada for Release of Product

Samples will undergo safety, identity, potency, dose and purity testing as part of the Health Canada product release procedure. Efforts will be made to try to achieve a minimum of $2.5x10^6$ viable CD34 cells/kg. This includes testing for viability, microbiology, mycoplasma and endotoxin.

Viral copy number (indicator of transduction efficiency) and α-Gal A activity will be assessed in the Medin laboratory. An aliquot of the drug product will be collected prior to cryopreservation. This aliquot will be cultured for 2 days in stem cell growth media and an α-Gal A assay will be performed to document expression and activity of the transgene product.

Only those approved by Health Canada will be released and transported to site for administration. Cell products that do not receive Health Canada approval will be discarded or donated for research as per the patient's wishes on the informed consent form. **Patients with transduced cell products that do not receive Health Canada approval may be re-mobilized again upon discussion and agreement with CTSC.**

6.4.3. Phase 3: Treatment Phase - Transplant (Days -2 to +12)

A) Day -2

Patients with a Health Canada approved transduced autologous CD34+ cell product will visit the Regional Stem Cell Transplant Centre on day -2 for blood work. Routine blood work will be repeated to ensure patient can be administered with high dose Melphalan. Refer to Study Calendar. If on day-2, there are significant Fabry Disease related changes in the patient's health status (health jeopardized with proceeding with transplant), a discussion may be warranted with the Clinical Trial Steering Committee to decide on whether or not patient can continue onto the trial.

B) Day -1: Melphalan Administration

After enrollment onto the trial, eligible patients will be administered a single dose of Melphalan I.V. at 100 mg/m^2 on Day -1 as per institutional procedures.

C) Day 0: Health Canada Approved Transduced Autologous $CD34^+$ Cell Product for Infusion

Transduced cells will be infused at the bedside on Day 0 according to the Infusion Manual.

D) Day 5: Neupogen® (filgrastim) Injection

During the Transplant phase, Neupogen® will be administered subcutaneously on day 5 or another day as per physician's discretion, at a dose of 5µg/kg until neutrophil count is >1.5x10^9/L in transplant phase, then filgrastim can be stopped.

E) Day 13-27: Please refer to Study Calendar.
After day +12, patients may be managed as outpatient or admitted if there are ongoing AEs that require close monitoring at the discretion of the treating physician. Assessments will be done as clinically indicated, frequency and duration of monitoring done at the discretion of the treating physician.

Re-infusion of Back-up Autograft of Non-Transduced, Non-CD34+ Selected Cells

The re-infusion of back-up autograft of non-transduced, non-CD34+ selected cells will occur under the following circumstances:

1) If a patient receives Melphalan and goes off study prior to receiving protocol treatment (transplant/autologous CD34+ cells expressing alpha Galactosidase A), patient will be re-infused the back-up autograft of non-transduced, non-CD34+ selected cells as per SOC and institutional policies. Assessments and monitoring of the patient will be performed as per SOC. Refer to Study Calendar in section 5.

2) Failure to Engraft

Failure to engraft is defined as absolute neutrophil count (ANC) < 0.2 × 10^9 cells/L or platelet count <10 x 10^9/L on or after day 21 of Treatment Transplant phase. If this occurs, a discussion will take place between the CTSC members to decide on whether or not the back-up autograft of non-transduced, non-CD34$^+$ selected cells comprising 2.5 × 10^6 CD34$^+$ cells/kg (which was collected at Phase 3 Mobilization) should be infused into the patient.

This event will be considered a SAE – refer to Section 10. The reason for failure to engraft will be investigated and meeting (s) held between CTSC members to decide on whether the trial will continue enrollment or be held. All decisions will be documented.

6.4.4. Phase 4: Post-Treatment Follow Up for 5 Years at Regional FD Centre

6.4.4.1. Enzyme Replacement Therapy Resumes

All patients in Canada with FD who receive ERT are required to meet the Canadian FD Treatment Guidelines to start (http://www.garrod.ca/wp-content/uploads/Canadian-FD-Treatment-Guidelines-2016.pdf

accessed Nov 28, 2016). Subjects in this study receive ERT with agalsidase alfa or agalsidase beta IV every 2 weeks and are evaluated in FD clinics every 6 months. Testing for anti-agalsidase antibodies including neutralizing antibodies is done every 6 months.

Patients will stop ERT at day 28 of Pre-Mobilization phase (at least 30 days prior to D0 of Transplant phase) and resume ERT at approximately Day +30 in Phase 4: Post-Treatment Follow up Phase. Thereafter ERT will be given every 2 weeks. For subjects who were on investigational agent agalsidase-alfa prior to SCT, they will be required to switch to agalsidase beta 1.0 mg /kg every 2 weeks at Day+30. This switch to agalsidase beta will not be required once agalsidase alfa becomes a marketed product. The total number of ERT infusions missed due to SCT will be 3 to 4 only depending upon the scheduling of the SCT. These missed doses are unlikely to cause any clinical deterioration in subjects with Fabry disease. There can be a carry over effect of ERT in some patients that can last for several weeks or months before symptoms return. During the shortage of Fabrazyme between 2009 and 2012 there was extensive experience with Fabry patients on low dose therapy; a return of neuropathic pain was the most common problem[42].

6.4.4.2. Enzyme Replacement Therapy may be stopped after 6 months post-transplant

Withdrawal of exogenous ERT will be considered in patients who are medically stable 6 to 18 months post autologous CD34+ cell transplant to determine the following:

1. Stability of plasma and leukocyte alpha-galactosidase activity levels.
2. Patient health status with emphasis on renal (urinalysis, proteinuria, eGFR), cardiac (EKG, holter monitor, echocardiogram, cardiac MRI) and neurologic (brain MRI or CT) routine investigations.
3. Stability of various metabolites in both plasma and urine (Gb3 and lysoGb3 and respective analogs).

All patients considered for withdrawal of exogenous ERT must meet the following criteria prior to stopping ERT:

1. The patient continues to make his own alpha gal-A enzyme at 6 months post-transplant as determined in the study reference laboratory at a level deemed acceptable by the investigator.
2. The patient is deemed medically stable by their study physician
3. The patient provides consent for withdrawal of ERT following discussion with their study physician, and this has been documented by the physician and/or nurse that is was discussed with the patient and they are in agreement.
4. The CTSC unanimously agree and provide written approval that ERT may be stopped for the patient after reviewing all relevant data and literature, and evaluating the risks vs. benefits to the patient.

All decisions made will be documented and filed along with their rationale. Close monitoring of these patients will be performed as per Table 3. Should any of these patients show any safety concerns after stopping ERT, ERT may re-start as per investigator's discretion. Refer to section **Error! Reference source not found.**.

6.4.4.3. Post-Transplant Management

Patients will be managed according to the SOP for autologous bone marrow transplant (ABMT) at the Regional Stem Cell Transplant Centre. Refer to the Study Calendar.

6.5. Treatment Delays

If at any point in stem cell mobilization or transplant treatment phase, the treating physician considers patient not appropriate to proceed with the transplant, transplantation may be withheld or delayed until patient recovers or patient may also be withdrawn from the study. Discussions with the CTSC members may occur in these circumstances and the reasons for withholding or delaying the transplantation or withdrawal of the patient will be documented.

6.6. Concomitant Medications

6.6.1. <u>Supportive Therapy and Permitted Concomitant Medications</u>

Standard supportive therapies for optimal medical care will be given throughout the study. This will be based on the attending physician as well as local institutional guidelines. Administration of antiemetics will be required. All concomitant medications and relevant supportive therapy must be recorded on the specified electronic case report forms (eCRF). All blood products are to be administered as per local institutional policy at the Regional Stem Cell Transplant Centre. The use of antibacterial, antifungal, and anti-viral agents are recommended according to local policy.

Fever, (defined as >38°C ×2 or 38.5°C ×1) in the setting of neutropenia (ANC <0.5 × 10^9/L) is a well-known life-threatening complication of transplant therapy. Empiric antibiotic administration consisting of broad-spectrum coverage is recommended as per institutional guidelines. Antifungal treatment is to be considered for the persistence of fever or emergence of new fever after 3 days of antibiotic administration. Serum galactomannan antigen enzyme-linked immunosorbent assay (ELISA) and high resolution CT scanning should be performed as clinically indicated. Prophylactic use of colony-stimulating growth factors is permitted.

6.6.2. <u>Prohibited Concomitant Medications</u>

ERT will be stopped after the last dose on day 28 of Pre-Mobilization phase (at least 30 days before ASCT on day 0 in Phase III). ERT is prohibited within 30 days prior to receiving ASCT on day 0 of the Transplant stage. ERT will resume at Day+30 post-transplant.

6.7. Premature Withdrawal/Discontinuation of Study Participants

Note that patients may discontinue from the study in the following instances:

a) Evidence of rapid organ dysfunction

b) Concurrent illness that, in the judgment of the Investigator, would affect assessments of clinical status to a significant degree and requires discontinuation of protocol therapy

c) Unacceptable toxicity

d) Request by the patient
e) Non-compliance with study protocol (e.g. failure to complete pre-transplant evaluations)
f) Insufficient number of cells are collected during leukapheresis
g) Patients who are positive for HBV, HCV, HIV, HTLV-1 or VDRL
h) Insufficient number of vector transduced cells or cells not suitable for re-infusion
i) Certificate of Analysis for cell product not approved by Health Canada

An exit visit will be conducted as per Study Calendar for patients who receive G-CSF and goes off study prior to receiving transplant. If a patient goes off study after receiving the transplant, subsequent assessments and follow up will be done as per Study Calendar unless the patient withdraws from any further study follow up.

If a patient receives Melphalan and goes off study prior to receiving protocol treatment, patient will be re-infused the back-up autograft of non-transduced, non-CD34+ selected cells as per SOC and institutional policies. Assessments and monitoring of the patient will be performed as per SOC.

Ozmosis Research should be informed immediately as soon as a patient is discontinued from the study.

6.8. Subjects Compliance and Dropout

Patients who withdraw from the study for reasons unrelated to therapy (re-infusion of transduced autologous cells), such as non-compliance or withdrawal of consent, will be considered dropouts. All such patients are still evaluable for toxicity and efficacy if they actually received the transplant.

Patients who withdrew from study for any reasons, prior to receiving the transplant will be replaced and will not be evaluable for safety and efficacy.

Patients who withdraw from the study due to adverse events related to the transplant (re-infusion of transduced autologous cells) will be considered as discontinuations. These patients will also be included in safety and efficacy assessments.

6.9. Contraception

Patients must be willing to abstain from sexual activity or willing to use double-barrier method during sexual intercourse from day of Melphalan administration until 12 months follow-up post-transplant. Sexually active male patients will use condoms and in addition, should ask their female partners with child-bearing potential [defined as a sexually mature woman who has not undergone hysterectomy or who has not been naturally postmenopausal for at least 24 consecutive months (i.e. who has had menses any time in the preceding 24 consecutive months)] to use oral, implantable or injectable contraceptives, contraceptive patch, intrauterine device, diaphragm with spermicidal gel when having sexual intercourse.

Patients must not donate sperm after receiving Melphalan in this study. Sperm banking will be recommended to any patient who would like to father children in the future. Refer to section 7 below for risks.

7. RISKS VS. BENEFITS

Please refer to risks in the current Product Monographs/Investigator Brochures and Informed Consent Form.

7.1. Benefits

This study is primarily designed as a safety study. The benefit of this single administration treatment may be to restore, or partially restore, α-gal A enzyme activity in adult males with FD. While it could reduce some of the symptoms of FD, this is unlikely in patients already taking regular dose ERT every 2 weeks. Whether sufficient restoration of α-gal A enzyme activity could occur such that exogenous ERT could be stopped is unknown.

7.2. Risks Due to Stem Cell Mobilization

G-CSF (Refer to Neupogen Product Monograph for details on risks)

a. Musculoskeletal: Myalgia. In clinical trials, medullary bone pain was the only consistently observed event in approximately one quarter of patients. Bone pain was mostly mild-to-moderate and easily controlled with non-narcotic analgesics. Uncommonly, narcotic analgesics were required.

b. Cardiovascular: Fluid retention (rare), transient hypotension, pericardial effusion

c. Dermatological: Local inflammation at injection site, rarely cutaneous vasculitis

d. Other: Transient mild to moderate elevation of uric acid, LDH, ALP, WBC, allergic reaction, fever and chills, headache, transient splenomegaly

e. There are very rare cases where patients developed leukemia years after use of G-CSF. It is unclear if there is a direct causal effect, but a link is reported.

7.3. Risks of Stem Cell Collection/Apheresis

Precautions are taken to avoid conditions such as hypotension, electrolyte abnormalities, thrombocytopenia, and coagulation abnormalities.

Rarely, complications arise with venous access and can lead to local bleeding, infection and depending on route, very rarely to pneumothorax, or hemothorax.

7.4. Risks of ex vivo Cell Transductions

There is no risk to the patient during the *ex vivo* transduction of the cells.

7.5. Intensive Therapy and Stem Cell Infusion

Melphalan is well tolerated and all of the Regional Stem Cell Transplant Centres have extensive experience with this agent post-transplant.

Melphalan is virtually unique in its use as a stem cell transplantation intensive regimen for non-malignant conditions such as systemic amyloidosis[43]. The Toronto Stem Cell Transplant Program data for multiple myeloma stem cell transplantations from 2000 to 2008 among mostly middle-aged to older patients receiving single agent melphalan at 200 mg/m^2 but with a small minority with dose reductions to 140mg/m^2 based on comorbidities or poor functional status revealed 14 non-relapse deaths among 843

consecutive transplant recipients or 1.7%.

Although there is no experience in using stem cell transplantation for patients with FD, lack of prior exposure to cytotoxic agents suggests that hematological recovery is likely to be rapid. Nevertheless, high-dose single agent melphalan therapy is associated with side-effects, including prolonged cytopenias and a treatment-related mortality of 1.7% in patients with multiple myeloma, all of whom have received prior chemotherapy and many of whom are more than 60 years of age. Other reported side effects include mucositis, alopecia, allergic reactions, skin hypersensitivity and, rarely, pulmonary complications. Sperm production may be reduced or stopped in men. Refer to Melphalan product monograph for all risks. Moreover, although adverse effects occur, this agent is among the best tolerated of any cytotoxic drug used in stem cell transplantation[44].

Stem cell infusion can cause tightness in chest, hypotension, coughing, chest pain, less urine output, weakness, hypersensitivity reactions, electrolyte disturbances, and rarely, engraftment syndrome (fever, rash, non-cardiogenic pulmonary edema).

7.6. Lentivirus Vectors (LVs)

LVs raise specific safety, ethical, and public health concerns. These include:

- Possible generation of RCL caused by the recombination of vector plasmids during the vector production process or by mobilization of proviral DNA *in vivo* by infectious retroviruses (i.e. HIV)
- Possible insertional mutagenesis, potentially leading to tumour formation
- Possible germline alteration and transmission of transgene to offspring
- Possible dissemination of the LV beyond the patient

Second-generation LV vector (replication incompetent - tested by IUVPF; 3′LTR SIN Design) will be used. For LV production, a triple transfection method that requires three plasmids, the envelope plasmid pMDG, the packaging plasmid R8.91 CMV, and our gene transfer plasmid (LV- hum.co.α-gal A), will be employed by IUVPF according to their SOPs and Master File.

7.7. Anti- α-gal A Antibody Development

Another potential risk is the development of anti-α-gal A antibodies due to the presence of the normal and/or corrective, but foreign, enzyme. The majority are IgG antibodies as measured by enzyme linked immunosorbent assay (ELISA). IgE antibody development has only been associated with use of agalsidase beta and is quite rare, with only 6 cases reported; anaphylaxis has not been reported to date[45]. The prevalence of IgG antibodies is between 20 and 56% in males on agalsidase alfa and 73-91% on agalsidase beta[46]. These antibodies have been associated with a higher risk of IAR, as well as increased levels of both Gb_3 and lyso-Gb_3 in urine and/or plasma[47,48,49]. *In vitro* neutralization of α-gal A has been recognized in up to 50% of males on ERT[48]. A recent retrospective cohort study suggests a significant reduction of the clinical benefit of ERT by the presence of neutralizing antibody in men with Fabry disease with worsening clinical parameters and symptoms[50]. This observation needs to

be confirmed in future studies with more patients and longer follow up. The presence of IgG against agalsidase beta is also associated with a higher risk of IAR[49].

Both the lower levels of continuous enzyme production and the immunosuppressive action of Melphalan may mitigate the risk of anti-α-gal A antibody formation as compared with the current regimen of ERT given biweekly as a large bolus. Transduction of more primitive hematopoietic cells may also lead to the development of tolerance by immature antigen presenting cells (APC) that are derived from the transduced primitive hematopoietic stem/progenitor cells. Nonetheless, it is not known whether antibodies will be produced against α-gal A following infusion of vector-transduced autologous cells and whether those antibodies will be neutralizing *in vitro* or *in vivo*. Testing for IgG antibody against α-gal A will be done as per Study Calendar. Patients will be closely monitored for any reactions due to the presence of anti-α-gal A antibodies.

7.8. Enzyme Replacement Therapy

All ERT will be given to all study subjects for the duration of this protocol as per the frequency in the study calendar. All subjects in this study will receive ERT with standard dose agalsidase beta 1.0 mg/kg or agalsidase alfa 0.2 mg/kg given I.V. every 2 weeks through the screening, and Pre-Mobilization phase of this study. ERT will be withheld as of day 28 of Pre-Mobilization phase (at least 30 days prior to day 0 of the ASCT) and resumed at Day 30+ post-transplant. Fabry patients tend to show some carryover effect of drug for some weeks after ERT is stopped. The three or four missed doses of ERT are very unlikely to have any short- or long-term consequences for the health of the study subjects. Agalsidase beta will be administered as per the product monograph (see section 0). Agalsidase alfa will be administered according to the Investigator's Brochure as these patients are all enrolled in the REP-081 study of ERT in Canada until agalsidase alfa becomes a marketed product, in which case, the product monograph will be followed. Agalsidase beta will be restarted at the same dose and dosing frequency at approximately 30 days post-transplant. If agalsidase alfa is not yet a marketed product, those patients who were taking investigational agent agalsidase alfa pre PBSCT will be switched to agalsidase beta 1.0 mg/kg given I.V. every two weeks at the 1 month post-transplant follow up visit. No switch to agalsidase beta is required once agalsidase alfa becomes a marketed product. Routine prophylaxis will be given with acetaminophen 325 mg two tablets p.o. taken 30 minutes pre each ERT infusion. In the event of a subject experiencing a serious IAR while at the Regional Stem Cell Transplant Centre outside the D-30 to D +30 period, the PI will consult with the subject's Fabry specialist who is also a member of the FACTS study team.

Patients who meet the criteria for stopping ERT administration post-transplant may discontinue treatment with ERT (See section 6.4.4.2). Potential benefits of stopping exogenous ERT post-transplant include avoidance of unnecessary treatment, avoidance of intravenous infusions every 2 weeks with patient discomfort, inconvenience with risks of local infection and bruising, decreased treatment cost, and decreased exposure to a drug that may cause anti-drug antibodies and IAR. By removing ERT as a potential antigen, anti-drug antibodies and IAR may diminish or disappear. As the presence of anti-drug antibodies has been associated with poorer

patient outcomes and increased biomarker levels in plasma and urine, stopping exogenous ERT could result in patient improvement over the long term[51,52,53].

Potential risks of stopping exogenous ERT post-transplant include decreased delivery of alpha-galactosidase enzyme to internal organs such as heart and kidneys and therefore decreased treatment of Fabry disease. While the plasma and leukocyte alpha-galactosidase activity levels are normal to high post-autologous transplant of CD34+ cells transduced with the lentiviral vector, the delivery and activity of alpha-galactosidase enzyme in other tissues and organs is as of yet unknown in this study. Withdrawal of ERT in patients on ERT only is known to be associated with a gradual return or worsening of Fabry disease symptoms (e.g. fatigue, diarrhea, neuropathic pain after 2-4 months)[54, 55]. Changes in organ function (e.g. heart and kidney) occur slowly over months to years[56]. By continuing to monitor Fabry patients closely with clinical evaluations and testing every 6 months as per Table 3, deterioration of disease status can be determined. If a patient shows any safety concerns including evidence of disease progression after stopping ERT, resumption of ERT or other specific Fabry disease therapy (e.g. chaperone therapy) will be offered to the patient as per standard of care. Additionally, psychological stress could be induced by making changes in therapy for this chronic condition. Once identified, this problem can usually be resolved through patient education and further discussions with the study physician and coordinator. Referral to a psychologist can be made as necessary.

The risks and benefits of participating in this study, including the potential benefits and harms of discontinuing exogenous ERT post-transplant, are outlined in the ICF and will be updated in the ICF as new information becomes available.

7.8.1. IARs (Infusion-Associated Reaction)

Most IARs are mild, short-lived, and occur during or in the first hour post-ERT infusion. IARs can include chills, fever, hives, muscle pain, dyspnea, nausea, vomiting, hypotension, paresthesiae, flushing, headache, fatigue, pruritus, pain in extremities, hypertension, chest pain, throat tightness, abdominal pain, dizziness, tachycardia, nasal congestion, diarrhea, and peripheral oedema. The usual onset is in the first 6 months after the start of ERT. IARs are usually managed easily by interruption or slowing of the infusion rate, along with use of oral antipyretics and antihistamines. Rarely are oral or parenteral corticosteroids required. As patients in this study will have already been receiving ERT a number of 6 months prior to transplantation, it is not expected that they will have any new or increased risks of IAR prior to the PBSCT. Post PBSCT, the subjects switched to agalsidase beta will have a slightly increased risk of IAR and the development of anti-agalsidase antibodies. The immunosuppressive effect of melphalan could theoretically reduce the risk or severity of IARs and the risk of the development or titre of anti-agalsidase antibodies. Appropriate treatment will be available to any subject who develops an IAR with agalsidase infusion at all of the regional Fabry Treatment Centres. These enzyme infusions and treatment of any IARs will be supervised by co-investigators who are also Fabry specialists.

8. STUDY INTERVENTION / INVESTIGATIONAL PRODUCT

8.1. Investigational Product

GMP-grade LV vectors will be produced and tested under GMP conditions at the Indiana University Vector Production Facility (IUVPF). The IUVPF has made and validated many LV preps for Clinical Gene Therapy Trials in the USA and Europe.

The purification of Fabry patient CD34+ cells will be done at the Juravinski Cancer Centre (see section 6.4.2.4).

The preparation and transduction of autologous Fabry patient CD34+ cells expressing α-Gal A will be done at the GMP compliant cell-processing facility Philip S. Orsino Facility at Princess Margaret Cancer Centre.

Each Fabry gene therapy product is autologous and thus patient-specific. Each patient product will be generated according to the SOPs on file at Dr. Jeffrey Medin's lab in Toronto. There will be no pooling of cells from different patients. As part of the Health Canada product release procedure, a minimum of 2.5×10^6 viable CD34 cells/kg is required. There is no maximum number of transduced CD34+ cells. All autologous Fabry patient cells expressing α-Gal A will be infused into the patient.

Note that the only investigational drug substance in the final drug product are autologous Fabry patient cells expressing αGal A. The drug product is otherwise composed of components that are all approved for intravenous infusion and are all of USP standard.

8.2. Preparation, Transport and Labelling

After leukapheresis, the apheresis product will be transported between 2-10°C from the participating site to Juravinski Hospital in Hamilton for $CD34^{+}$ selection (refer to Apheresis Product Transport Manual). After CD34+ selection, the enriched CD34 product will be transported between 2-8°C from Juravinski Hospital to Philip Orsino Facility for lentiviral transduction. As soon as the enriched CD34 product arrives at the Orsino Facility, the cells will be removed, placed in growth media and incubated at 37°C until the final washes for cryopreservation are performed.

The drug product is prepared by Dr. Jeffrey Medin's staff at the Philip Orsino Facility by a single step dilution of the drug substance (Fabry patient cells expressing αGal A resuspended in CryoStor media) into a sterile Cryostore freezing bag. The drug product is then frozen under vapour phase liquid nitrogen (-144 to -199°C) while sterility analysis is completed and clearance from Health Canada is obtained.

Once Health Canada clearance has been obtained, the Cryostore is transported by a certified courier in vapour phase liquid nitrogen to the local stem cell centre where the infusion will occur. The final product is kept in vapour phase liquid nitrogen until it is ready for infusion into the patient. The infusion bag is then thawed and cells are transplanted to the recipient patient as described in the Infusion Manual. Transfer of the product from the courier to the clinical staff will be documented on Product Transfer Forms.

The final product in a transfusion bag to be delivered to the patient will be labeled with the unique patient ID code and as per the Infusion Manual.

8.3. Drug Product Accountability

The patient's cells will be tracked from the time of mobilization to when they are infused into the patient. The samples will be tracked by a unique identifier. An Accountability Form will be completed and available for review by study monitors during monitoring visits and close out visit.

8.4. Stability

All investigational products must be kept in a secure place under appropriate storage conditions. The stability studies performed suggest that the transduced Fabry cells expressing α-Gal A are stable for 6 hours when maintained at ambient temperature in CryoStor media.

8.5. Drug Product Administration

The infusion instructions for the drug product and precautions will be detailed as per the Infusion Manual and applicable institutional SOPs.

9. CRITERIA FOR MEASUREMENT OF STUDY OUTCOMES AND STUDY ENDPOINTS

9.1. Primary Endpoint

Toxicity will be assessed using the National Cancer Institute of Canada (NCIC) Common Terminology Criteria for Adverse Events (CTCAE), Version 4.03 (See Appendix II). Safety measurement will be based on all clinical and laboratory assessment post-baseline. The assessment will be the frequency of clinically notable abnormal vital signs and laboratory values, and the frequency of treatment-related adverse events. No formal statistical tests will be performed in the safety evaluation.

The reason for discontinuation will be listed for patients who fail to complete the study.

9.2. Secondary Endpoints: Assessment of Functional Efficacy Measurements

The following laboratory measures of efficacy will be determined:

- Increase in α-gal A enzyme activity within the plasma, leukocytes, and BM
- Reduction of Gb_3 in plasma and urine
- Reduction of lyso-Gb_3 and related analogues in plasma and urine
- Persistence of LV-transduced cells as measured by quantitative (q)PCR
- Measure the transduction efficiency of hematopoietic stem/progenitor cells

All samples will be collected and stored according to the lab manual.

9.2.1. <u>Increase in α-Galactosidase A Activity</u>

Briefly, α-gal A activity will be measured in plasma, isolated leukocytes, bone marrow, and nucleated BM cells using standard methods in an accredited laboratory under the

supervision of Dr. Tony Rupar at LHSC. Although patients will continue to receive their usual I.V. ERT every 2 weeks as a safety issue until 30 days prior to day 0 of Transplant stage, the plasma α-gal A activity rapidly returns to baseline within 24 hours due to rapid uptake of exogenous agalsidase beta by the reticuloendothelial system and other organs. An increase in plasma α-gal A activity above baseline thereafter should indicate an increase in enzyme production from transduced cells.

9.2.2. Globotriaosylceramide (Gb3) levels

Tandem mass spectrometry (MS/MS) analyses of Gb_3 levels in plasma and urine will be performed as described in the Investigators Brochure[57]. A consistent change in the levels of Gb_3 in plasma and urine as substrate for the enzyme should reflect increased or decreased α-gal A activity from baseline status. Daily sampling will be done to determine the onset of the biological effect of gene transfer while hospitalized. Post-discharge, samples will be collected for assay at each clinic visit to study completion.

9.2.3. Levels of Globotriaosylsphingosine (lyso-Gb3) and Related Analogues

Tandem mass spectrometry analyses of lyso-Gb_3 levels in plasma and urine will be performed as described in the Investigators Brochure [57,58,58,59.] A consistent change in the levels of lyso-Gb_3 and related analogues in the plasma and urine should reflect increased or decreased α-gal A activity from baseline status. Daily sampling will be done to determine the time course of the biological effect of gene transfer while hospitalized. Post-discharge, samples will be collected for assay at each clinic visit to study completion.

9.2.4. Methyl-cellulose PCR Assay

The methyl-cellulose PCR assay is a way to measure the efficiency of proviral integration into committed progenitor cells that are presumably derived from the transduced hematopoietic stem cells. It is an old assay but still used in the field. Patient CD34+ cells, from the transductions, are plated into methyl-cellulose with growth factors at limiting dilutions. After about 2 weeks, isolated colonies are picked and assayed by PCR for the presence of integrated provirus into the colony population.

9.3. Secondary Safety Measurements

9.3.1. Generation of Replication Competent Lentiviruses (RCL)

The possible generation of RCL in LV-based gene therapy trials is a safety concern. Note that the first clinical gene therapy trial involving recombinant LV was in HIV patients with high wild-type HIV loads. No recombination between the therapeutic LV used to transduce the T cells and wild-type HIV - as evidenced by the lack of vesicular stomatitis virus glycoprotein (VSV-g) antibody or VSV-g DNA sequence measured by sensitive PCR assays - were observed in that study[15]. RCL could also occur during manufacture of LV vectors. Second-generation LV (replication-incompetent with 3′ SIN LTRs) will be used in this trial. In this system, five of the nine HIV-1 genes are eliminated, leaving the gag and pol reading frames, which encode for the structural and enzymatic components of the virion, respectively, and the tat and rev genes, fulfilling transcriptional and post-transcriptional functions[59]. Testing for RCL during production is an integral part of the

release criteria of the GMP-grade virus from the IUVPF. Further, detailed testing by Dr. Cornetta on 30 independent GMP-grade LV preparations from his institution and from other GMP facilities has not shown any evidence whatsoever of RCL production[60].

9.3.2. Vector copy number & persistence by quantitative real-time PCR

Vector copy number and persistence analysis will be performed on leukocytes isolated from peripheral blood and bone marrow aspirate. The Woodchuck hepatitis virus post-transcriptional regulatory element (WPRE) sequence present in our LV backbone will be used as a marker sequence for qualitative genomic PCR and real-time qPCR analysis of vector copy number and persistence as described in the Investigators Brochure.

9.3.3. Anti-agalsidase A Antibody Level Determination

The Medin lab will use an in-house ELISA to measure anti-agalsidase IgG.

9.4. Correlative Studies

9.4.1. Mandatory Correlative Studies

For each patient, urine and plasma specimens for the analysis of Gb3 isoforms/analogues and lyso-Gb3 and analogues will be collected as per the Study Calendar. Please refer to the Lab Manual for collection, processing and shipment details.

9.4.1.1. Analysis of Gb_3 isoforms/analogues in urine and plasma

The relative quantification of Gb_3 isoforms/analogues will be performed in urine and plasma for patients enrolled in this study. The increased alpha-galactosidase A activity, due to gene therapy, is expected to correlate with a decrease of Gb_3 isoforms/analogues levels. Analysis will be performed according to the standard operating procedure (SOP)[57]. Analysis of Gb_3 isoforms/analogues in urine and in plasma will be done by tandem mass spectrometry.

9.4.1.2. Analysis of lyso-Gb_3 and analogues in plasma

The method for the quantification of lyso-Gb_3 and its 6 analogues in plasma is detailed in the SOP[52,59]. The molecules are analyzed by tandem mass spectrometry.

Analysis of lyso-Gb_3 and analogues in urine

The method for the relative quantification of lyso-Gb_3 and its analogues in urine is detailed in SOP[51,58]. The molecules are analyzed by tandem mass spectrometry. The levels of lyso-Gb_3 and its analogues will be used as an indication of the gene therapy efficiency.

9.4.2. Optional Correlative Studies

9.4.2.1. Optional metabolites studies in urine

Galabiosylceramide (Ga_2) isoforms/analogues are another group of Fabry disease metabolites recently discovered[61]. These metabolites will be analyzed using tandem

mass spectrometry in urine from gene therapy patients. We will use remaining samples from the mandatory studies for these optional metabolites studies.

9.4.3. Optional Research Studies

Additional optional research samples after 1 month follow up visit (above what is already required in Table 2 above) may be taken for research at timepoints determined by the CTSC if patient consents and if deemed clinically or scientifically relevant by CTSC:

a) **Bone marrow aspirate:** alpha Gal-A activity will be examined as per section 9.2.1 and vector copy number and persistence analysis will be performed on the bone marrow aspirate as per section 9.3.2. A methylcellulose assay will also be performed as in section 9.2.4.
b) **Skin biopsy:** alpha Gal-A activity will be examined (refer to section 9.4.3.1).
c) **Blood:** Gb3 & isoforms & analogues and Lyso-Gb3 and related analogues, Plasma α-Gal A activity, α Gal A activity in leukocytes, Anti-agalsidase antibodies assays, CBCs, qPCR (same tests done in section 9 using blood) will be examined. In addition, the clonality or integration will be examined using LAMPCR (refer to section **Error! Reference source not found.**).
d) **Urine:** Gb3 and isoforms & analogues and Lyso-Gb3 and related analogues, Ga2 isoforms and analogues per section 9.4 and podocytes as per section 9.4.3.2 will be examined.

Additional research tests may be performed on these optional samples if deemed clinically or scientifically interesting by the CTSC.

9.4.3.1. Alpha Gal –A Activity in Optional Skin Biopsy

Alpha Gal-A activity will be assayed in cultured fibroblasts obtained from a skin biopsy specimen. Skin biopsies can be collected in a minimally invasive manner and possible changes in the enzyme level in cultured cells from this surrogate organ can be assayed essentially as described for peripheral blood leukocytes.

9.4.3.2. Examining Clonality in Optional Blood Samples

Linear Amplification Mediated PCR (LAMPCR) will be used as the method of clonality analysis.

9.4.3.3. Examining Podocytes in Optional Urine Samples

Podocytes will be collected by centrifugation, examined by microscopy, and counted. The apoptotic status of the 'shed' cells may also be measured by standard apoptosis assays.

10. SAFETY AND REPORTING REQUIREMENTS

10.1. Adverse Event

An **adverse event** (AE) is any untoward medical occurrence in a clinical investigation subject administered a pharmaceutical product (for this study, the re-infusion of the transduced autologous cells) during the course of a study and which does not necessarily have to have a causal relationship with this treatment. An adverse event can therefore be any unfavorable and unintended sign (including an abnormal laboratory finding), symptom, or disease temporally associated with the use of an investigational product, whether or not considered related to the medicinal product.

Disease signs, symptoms, and/or laboratory abnormalities already existing prior to the use of the product are not considered AEs after administration of the study product unless they reoccur after the subject has recovered from the pre-existing condition or they represent an exacerbation in intensity or frequency.

A laboratory test abnormality that is considered clinically relevant (e.g. causing the subject to withdraw from the study, requiring treatment or causing apparent clinical manifestations) or judged relevant by the Investigator should be reported as an adverse event.

10.2. Adverse Event Documentation

Adverse events will use the descriptions and grading scales found in the revised NCI Common Terminology Criteria for Adverse Events (CTCAE). This study will utilize the CTCAE Version 4.03 for adverse event reporting.

All AEs must be recorded on electronic case report forms (eCRFs). Documentation must be supported by an entry in the subject's file. Each event should be described in detail along with start and stop dates, severity, relationship to protocol treatment or study procedures (see section 10.6) as judged by the Investigator, action taken and outcome.

10.3. Serious Adverse Event

A **Serious Adverse Event or Reaction** is any AE occurring at any dose that:

- Results in death
- Is life-threatening
- Requires inpatient hospitalization or prolongation of existing hospitalization
- Results in persistent or significant disability/incapacity
- Is a congenital anomaly / birth defect
- Is an important medical event that may not be immediately life threatening or result in death or hospitalization, but may jeopardize the subject and may require medical or surgical intervention to prevent one of the outcomes listed above (example: intensive treatment in an emergency room or at home for bronchospasm, convulsions that do not result in hospitalization). Medical and scientific judgment should be exercised in deciding whether some events should be considered as serious because their quick reporting to the sponsor may be of interest for the overall conduct of the study.

Life-threatening: The term "life-threatening" in the definition of "serious" refers to an

adverse event in which the subject was at risk of death at the time of the event. It does not refer to an adverse event that hypothetically might have caused death if it were more severe.

Hospitalization: Any adverse event leading to hospitalization or prolongation of hospitalization will be considered as Serious, UNLESS at least one of the following exceptions are met:

- The admission results in a hospital stay of less than 12 hours.

OR

- The admission is pre-planned (i.e., elective or scheduled surgery arranged prior to day 1 of the study or for prophylactic insertion of a gastric feeding tube).

OR

- The admission is not associated with an adverse event (eg, social hospitalization for purposes of respite care).

However, it should be noted that invasive treatment during any hospitalization may fulfil the criteria of 'medically important' and as such may be reportable as a serious adverse event dependant on clinical judgement. In addition, where local regulatory authorities specifically require a more stringent definition, the local regulation takes precedent.

Disability means a substantial disruption of a person's ability to conduct normal life's functions.

Important medical event: Any adverse event may be considered serious because it may jeopardize the subject and may require intervention to prevent another serious condition.

Any death (regardless of cause except if death is due to PD) that occurs from the time of administration of the first dose of study therapy until 28 days after the administration of the study treatment and any death occurring after this time that is judged at least possibly related to the protocol treatment, will be promptly reported. Death due to PD does not need to be reported as a SAE.

All serious adverse events (SAE) must be recorded on eCRFs. In addition, all serious adverse events are subject to reporting using the SAE form and must be submitted to Ozmosis Research Inc.

Pregnancies occurring in study subjects/sexual partner(s) will be treated procedurally as SAEs. Pregnancies occurring in study subjects or their sexual partner(s) after study treatment should be reported separately on Pregnancy Report Form.

Any pregnancies must be reported to Ozmosis Research Inc. within 24 hours of being made aware of the event. The subject's sexual partner should be referred to an obstetrician/gynecologist experienced in reproductive toxicity for further evaluation and counseling.

Investigator /designee will follow up pregnancies until completion to assess the health of sexual partner and baby and promptly notify the Ozmosis Research Inc. of any additional

information related to the pregnancy within 24 hours. The Investigator will provide the outcome of the pregnancy (medical termination, live or still birth) or any additional significant event related to the pregnancy as a follow up to the initial pregnancy reporting form within 24 hours of his/her knowledge. All neonatal deaths that occur within 30 days of birth should be reported, without regard to causality, as SAEs. In addition, any infant death after 30 days that the Investigator suspects is related to the in utero exposure to the study treatment should also be reported.

Failure to engraft will be considered a SAE. Failure to engraft is defined as absolute neutrophil count (ANC) < 0.2×10^9 cells/L or platelet count <10×10^9/L on or after day 21 of Treatment Transplant phase. Refer to section 0.

10.4. Reporting Serious Adverse Events

All serious adverse events (SAE) defined as per ICH guidelines (see below) and other adverse events must be recorded on case report forms. In addition, all serious adverse events must be reported by using the SAE form and must be submitted to Ozmosis Research Inc.

Serious Adverse Event Reporting Instructions

All serious adverse events must be reported as follows:

Within 24 hours: Report initial information (on trial specific SAE report form) by fax or email to:

Ozmosis Research Inc.
Phone: 416-634-8300
Fax: 416-598-4382
Email: ozmsafety@ozmosisresearch.ca

The initial information should always contain:
- Name of Reporter/Investigator,
- Subject Identification,
- Adverse Event Term,
- Study Treatment Dose and Infusion Dates

On the next working day: Fax or email completed trial-specific Serious Adverse Event form

10.5. Procedure for Expedited Reporting

Responsibility for Reporting Serious Adverse Events to Health Canada

Ozmosis Research Inc. will provide expedited reports of SAEs to Health Canada according to applicable guidelines and regulations (including the 7-day notification for death and life-threatening events), i.e. events which are BOTH serious AND unexpected, AND which are thought to be related to protocol treatment (or for which a causal

relationship with protocol treatment cannot be ruled out).

Responsibility for Reporting Serious Adverse Events to CTSC & Medical Monitors

Ozmosis Research Inc. will be responsible for submitting all SAE reports received from the sites, to the CTSC & Medical Monitor within 24 hours of receipt.

Reporting Serious Adverse Events to Local Research Ethics Boards

Ozmosis Research Inc. will notify all Investigators of all Serious Adverse Events that are reportable to regulatory authorities in Canada from this trial to the Sponsor. This includes all serious events that are unexpected and related to protocol treatment. Investigators must notify their Research Ethics Boards (REBs) and file the report with their Investigator Site File. Documentation that serious adverse events (SAEs) have been reported to REBs must be kept on file at Ozmosis Research Inc.

Documentation can be any of the following:

- letter from the REB acknowledging receipt
- stamp from the REB, signed and dated by REB chair, acknowledging receipt
- letter demonstrating the SAE was sent to the board

All expedited serious adverse events occurring within a centre should also be reported to local REBs.

10.6. Adverse and Serious Adverse Events and Reporting Period

"Protocol treatment" in the context of this study is defined as (transplant/autologous CD34+ cells expressing alpha Galactosidase A) .

"Study procedures" in the context of this study refers to all study procedures such as apheresis, autologous transplantation procedure, blood draws, insertion of central lines, etc. as well as any protocol-specified drugs used during the procedures such as administration of G-CSF, Melphan, Plerixafor.

Attribution of an AE or SAE to protocol treatment or study procedures will be determined by the study investigator. Refer to section 10.7 below.

AE and SAE Reporting Period

From the date patient signs study informed consent to before patient receives protocol treatment on day 0, only the **AEs and SAEs *related* to the study procedures** will be reported on eCRFs.

All AEs and SAEs (related or unrelated to protocol treatment) will be reported after the

patient receives protocol treatment on day 0 until 30 days after protocol treatment or after 1 month follow up visit, whichever is later.

In addition, any known untoward event of any severity that occurs subsequent to the AE/SAE reporting period (30 days after protocol treatment or after 1 month follow up visit, whichever is later) that the Investigator assesses as at least possibly related to the protocol treatment (i.e., the relationship cannot be ruled out) should also be reported as an AE or SAE.

The investigator shall provide follow-up information as and when available in a new follow-up SAE form. All SAEs must be followed until resolved, become chronic, or stable unless the subject is lost to follow up. Resolution status of such an event should be documented on the CRF.

10.7. Relationship

For all AEs, relationship to protocol treatment or study procedures will be reported on the appropriate AE CRF page. The PI must judge whether the protocol treatment or study procedures caused or contributed to the AE in which case it is considered to be an ADR, and report it as either:

Related (definitely, probably or possibly): there is a reasonable possibility that the protocol treatment or study procedures caused or contributed to the AE; this conclusion may be supported by the following observations, though these are not required for the determination of relatedness:

- There is a plausible time sequence between onset of the AE and study treatment;
- There is a plausible biological mechanism through which study treatment may have caused or contributed to the AE;

Not related: It is highly unlikely or impossible that the protocol treatment or study procedures caused or contributed to the AE; this conclusion may be supported by the following observations, though these are not required for a determination of not related:

- another cause of the AE is evident and most plausible;
- the temporal sequence is inconsistent between the onset of the AE and the administration of protocol treatment or study procedures; a causal relationship is considered biologically implausible;

11. STATISTICAL CONSIDERATIONS

11.1. Study Design and Justification of Sample Size

This is a multi-centre, non-randomized, open-label, prospective pilot study of a novel stem cell gene therapy protocol in up to 6 adult male patients with confirmed FD currently receiving ERT. There is no formal statistics for the sample size determination.

After each patient completes the 1 month post-transplant follow-up visit, a safety review meeting will take place with the Clinical Trial Steering Committee (CTSC) (refer to section 6.1.2) and Ozmosis Research Inc. to review the safety data (refer to section 6.2). If there is no protocol treatment-related Serious Adverse Events (any SAEs including failure to engraft deemed by CTSC to be definitely, probably or possibly related to infusion of transduced autologous $CD34^+$ cells), or any significant safety concerns as defined in section 6.1.1, a decision will be made based on the data submitted for the patient regarding whether the study will be re-opened for the next patient to start treatment phase of the study. Only when it is deemed safe can the next patient start the treatment phase of the study.

11.2. Study Population

The study population will consist of all patients who are enrolled and received the re-infusion of transduced autologous cells (study treatment). All analyses will be conducted using the study population. Any patient who is registered on to this trial but never receives study treatment will be described, including the reason(s) for non-participation.

11.3. Statistical Analysis Methods

Tabulations and descriptive statistics will be employed in the analysis of primary and secondary objectives. Adverse events will be tabulated by National Cancer Institute of Canada Clinical Trials Group (NCIC CTG) Expanded Common Toxicity Criteria version 4.03. Additional summary tables will be generated for patients with serious adverse events, patients with related adverse events. Statistical analysis of laboratory observations will include comparison of means or medians by Paired T test, the Wilcoxon signed rank test and mixed model as necessary. Significance will be set at a 0.05 level. As the expected number of subjects is only up to 6, statistical analysis will be limited in power and the nature of the analysis is exploratory.

Any deviation from this statistics section of the protocol along with the accounting for missing, unused and spurious data will be described in the final report.

11.4. Evaluation of Safety

Terminology Criteria for Adverse Events (CTCAE) V4.03 will be used for grading of AEs. Investigators will provide their assessment of causality as 1) unrelated, 2) unlikely, 3) possibly related, 4) probably, or 5) definitely related. The results will be tabulated to examine their frequency, organ systems affected, severity, and relationship to study treatment.

11.5. Evaluation of Response

The following laboratory measures of efficacy will be determined:

- Increase in α-gal A enzyme activity within the plasma, leukocytes, and BM
- Reduction of Gb_3 in plasma and urine
- Reduction of lyso-Gb_3 and related analogues in plasma and urine
- Persistence of LV-transduced cells as measured by quantitative (q)PCR

12. ETHICS

12.1. Informed Consent

Subject / Legally acceptable representative (LAR) (as applicable) consent must be obtained according to local Institutional and/or University Human Experimentation Committee requirements prior to any study-specific screening procedures. It will be the responsibility of the local participating investigator to obtain the necessary clearance, and to indicate in writing to Ozmosis Research Inc. that such clearance has been obtained, before the trial can commence at that centre. Sample English consent forms for the trial will be provided. A copy of the initial full board REB approval and approved consent form must be sent to Ozmosis Research Inc. The subject/LAR must sign consent prior to registration.

12.2. Ethics Board Approval

Each participating centre will have on file with Ozmosis Research Inc., a list indicating the composition of its REB consistent with Canadian (applicable) regulatory guidelines. This list will be updated as appropriate.

For Canadian sites, a Health Canada REB Attestation Form must be completed and signed by the REB representative. Alternatively, an attestation may be included in the signed local ethics approval document. This documentation must be received by Ozmosis Research Inc. before the centre can be locally activated.

Initial approval: Site is required to obtain full board local ethics approval of the protocol and consent form by the appropriate REB prior to commencement of the clinical trial at the site.

Continuing approval: Annual (or as required by the REB) re-approval may be required for as long as subjects are being followed on protocol. It will be investigator’s responsibility to apply for and obtain the re-approval.

Amendment: All protocol amendments will be confirmed in writing and submitted, as appropriate, for review by the REB and health authorities. Amendments will be reviewed and approved by applicable regulatory authorities prior to central implementation of the amendment, and by REBs prior to local implementation, EXCEPT when the amendment eliminates an immediate hazard to clinical trial subjects or when the change(s) involves only logistical or administrative aspects of the trial.

REB Refusals: If an REB refuses to approve this protocol (or an amendment/revision to this protocol), Ozmosis Research Inc. must be notified immediately of the date of refusal and the reason(s) for the refusal. Notification will then be made to Health Canada.

Serious Adverse Events, Safety Updates and Investigator Brochure Updates: During the course of the study serious adverse events, safety updates or investigator brochure updates may be sent to you for reporting to your REB. If/when this occurs documentation of REB submission of this information must be forwarded to Ozmosis Research Inc.

13. DOCUMENTATION, RECORD ACCESS AND MAINTENANCE OF STUDY

RECORDS

13.1. Documentation of Subject’s Participation

A statement acknowledging the participation of a subject in this clinical trial must be documented in the subject's medical records along with the signed ICF.

13.2. Regulatory Requirements

The following documents are required:

- All Investigators must complete and sign the Health Canada Qualified Investigator Undertaking form. The completed forms must be returned to the Ozmosis Research Inc. prior to site activation.
- All applicable regulatory documents as listed in the Protocol Activation Checklist provided by Ozmosis Research Inc. to the sites.
- Ozmosis Research Inc. will submit via fax to Health Canada a completed Health Canada Clinical Trial Site Information Form after local activation of each participating Canadian centre.

13.3. Subject Confidentiality and Access to Source Data/Documents

Any research information obtained about the subject in this study will be kept confidential. A subject will not be identified by name, only by his/her initials. The subject’s name or any identifying information will not appear in any reports published as a result of this study.

However, information obtained from individual subject’s participation in the study may be disclosed with his/her consent to the health care providers for the purpose of obtaining appropriate medical care. The subject’s medical records/charts, tests will be made available to Ozmosis Research Inc., the sponsor University Health Network, its potential eventual partners, the Canadian HPFB/TPD, the REB and any other regulatory authorities. This is for the purpose of verifying information obtained for this study. Confidentiality will be maintained throughout the study within the limits of the law.

A subject’s name will not be given to anyone except the researchers conducting the study, who have pledged an oath of confidentiality. All identifying information will be kept behind locked doors, under the supervision of the study Principal Investigator and will not be transferred outside of the hospital.

A subject may take away his/her permission to collect, use and share information about him/her at any time. If this situation occurs, the subject will not be able to remain in the study. No new information that identifies the subject will be gathered after that date. However, the information about the subject that has already been gathered and transferred may still be used and given to others as described above in order to preserve the scientific integrity and quality of the study.

13.4. Confidentiality of the Study

Data generated as a result of this study are to be available for inspection on request by local health authority auditors, the Sponsor’s Study Monitors and other personnel (as

appropriate) and by the REB. The Investigator shall permit sponsor, authorized agents of the sponsor, CRO and regulatory agency employees to enter and inspect any site where the protocol treatment or records pertaining to the protocol treatment are held, and to inspect all source documents.

All information disclosed or provided by the Sponsor, or produced during the clinical trial, including, but not limited to, the clinical trial protocol, the Investigator's Brochure, the CRFs, manuals, and results obtained during the course of the clinical trial, is confidential. The regional co-Investigator at each participating site and any person under his/her authority agrees to undertake to keep confidential and not to disclose the information to any third party without the prior written approval of the Sponsor. This excludes the required REB submissions.

13.5. Registration of Clinical Trial

Prior to the first subject being registered/enrolled into this study, the Sponsor will be responsible for ensuring that the clinical trial is registered appropriately to remain eligible for publication in any major peer-reviewed journal, adhering to the guidelines put forth by the International Committee of Medical Journal Editors (ICMJE).

13.6. Data Reporting and Data Management

The data will be collected on electronic CRFs during this study. All data for this trial will be analyzed using the Medidata Rave database. Data management will be conducted by Ozmosis Research Inc.

13.7. Case Report Forms

Electronic CRFs will be used for this study. This also applies to records for those patients who fail to complete the study. If a patient is withdrawn from the study because of a treatment-limiting adverse event, thorough efforts should be made to document the outcome clearly.

The completed eCRF should be reviewed, signed, and dated by the Investigator in a timely fashion.

Please see the study-specific eCRF completion guidelines which have been provided to your site by Ozmosis Research Inc. The timelines and details for submission of eCRFs are included in these guidelines.

13.8. Maintenance of Study Records

To enable evaluations and/or audits from Regulatory Authorities, Ozmosis Research Inc. or the Sponsor, the Investigator agrees to keep records, including the identity of all participating subjects (sufficient information to link records, CRFs and hospital records), all original signed informed consent forms, copies of all CRFs, source documents, and detailed records of treatment disposition. The Investigator should retain these records for 25 years after study close-out as required by Canadian regulations or as specified in the Clinical Trial Agreement, whichever is longer.

If the investigator relocates, retires, or for any reason withdraws from the study, then the Sponsor should be prospectively notified. The study records must be transferred to an acceptable designee, such as another investigator, another institution, or to the Sponsor. The investigator must obtain the Sponsor's written permission before disposing of any records.

14. QUALITY ASSURANCE AND QUALITY CONTROL

As per the Guidelines of Good Clinical Practice (CPMP/ICH/135/95), the sponsor will be responsible for implementing and maintaining quality assurance and quality control systems.

14.1. On Site Monitoring / Auditing

Ozmosis Research Inc. will organize on-site monitoring of this study to be conducted as per the Monitoring Plan.

As this trial is conducted under a CTA with Health Canada, your site may be subject to an inspection by the Health Products and Food Branch Inspectorate. Other audits may be conducted by the study sponsor and Ozmosis Research Inc.

For the purpose of ensuring compliance with the clinical trial protocol, ICH GCP and applicable regulatory requirements, the Investigator agrees to permit study monitoring/auditing by or on the behalf of the Sponsor and inspection by applicable Contract Research Organization (CRO) auditors, REB, and Health Canada staff. The Investigator agrees to allow the monitors, auditors or inspectors to have direct access to his/her study records, including source data/documents.

15. ADMINISTRATIVE PROCEDURES

15.1. Amendments to the Protocol

Modifications of the signed protocol are only possible by approved protocol amendments authorized by the Sponsor. All protocol amendments will be approved by the REB prior to implementation. The Investigator must not implement any deviation from, or change to the protocol, except where it is necessary to eliminate an immediate hazard to trial subject or when the change(s) involves only logistical or administrative aspects of the trial.

15.2. Protocol Deviations and Violations

All violations or deviations are to be reported to the site's REB (as per REB guidelines). All REB correspondence is to be forwarded to Ozmosis Research. The site must notify Ozmosis Research and/or sponsor immediately of any protocol violations.

15.3. Premature Discontinuation of the Study

The Sponsor reserves the right to discontinue the trial for any reason but intends only to

exercise this right for valid scientific or administrative reasons. After such a decision, the Investigators must contact all participating patients immediately after notification. Standard therapy and follow-up for subjects will be assured and, where required by the applicable regulatory requirement(s), the relevant regulatory authority(ies) will be informed.

The REB will be informed promptly and provided with a detailed written explanation for the termination or suspension.

As directed by the Sponsor, all study materials must be collected and all CRFs completed to the greatest extent possible.

16. LEGAL ASPECTS

16.1. Responsibilities of the Investigator

One Qualified Investigator (QI) (must not be an Attending, Residents or Fellows) will oversee the trial at each clinical centre. The QI undertakes to perform the study in accordance with this clinical trial protocol, ICH GCP Guidelines and the applicable national regulations and local REB requirements.

The QI may appoint other individuals as he/she deems appropriate to assist in the conduct of the study. All appointed designates will be listed in the site delegation log. The appointed designates will be supervised by and under the responsibility of the QI.

16.2. Publication Policies

Publication of the study results may only be allowed with written permission from the Project PI.

For publications, the order of authorship will be determined by the Project PI of the study. The order will be based on who have made the most significant contribution to the overall success of the study. This contribution will be assessed, in part but not entirely, in terms of patients enrolled and will be reviewed at the end of the trial by the Project PI.

17. Appendix I: ECOG Performance Status[62]

ECOG PERFORMANCE STATUS*	
**Grade**	**ECOG**
0	Fully active, able to carry on all pre-disease performance without restriction
1	Restricted in physically strenuous activity, but ambulatory and able to carry out work of a light or sedentary nature, e.g., light house work, office work
2	Ambulatory and capable of all selfcare, but unable to carry out any work activities. Up and about more than 50% of waking hours
3	Capable of only limited selfcare, confined to bed or chair more than 50% of waking hours
4	Completely disabled. Cannot carry on any selfcare. Totally confined to bed or chair
5	Dead

18. APPENDIX II: Common Terminology Criteria for Adverse Events

This study will utilize the NCI Common Terminology Criteria for Adverse Events Version 4.03 (NCI-CTCAE v4.03) for adverse events and serious adverse event reporting. A copy of the NCI-CTCAE v4.03 can be downloaded from the following website:

http://evs.nci.nih.gov/ftp1/CTCAE/CTCAE_4.03_2010-06-14_QuickReference_5x7.pdf

19. REFERENCES

1 .Levey AS, Stevens LA, Schmid CH, et. al. for the CKD-EPI (Chronic Kidney Disease Epidemiology Collaboration). A New Equation to Estimate Glomerular Filtration Rate. *Ann Intern Med*. 2009 May 5; 150:604-612.

2 .Desnick, R.J., Y.A. Ioannou, and C.M. Eng, *Galactosidase A deficiency: Fabry disease.*, in *The Metabolic and Molecular Bases of Inherited Diseases*, C.R. Scriver, et al., Editors. 2001, McGraw-Hill: New York. p. 3733-3774.

3. Eng, C.M., J. Fletcher, W.R. Wilcox, S. Waldek, C.R. Scott, D.O. Sillence, F. Breunig, J. Charrow, D.P. Germain, K. Nicholls, and M. Banikazemi, *Fabry disease: baseline medical characteristics of a cohort of 1765 males and females in the Fabry Registry*, in *J Inherit Metab Dis*2007. p. 184-92.

4. Eng, C.M., N. Guffon, W.R. Wilcox, D.P. Germain, P. Lee, S. Waldek, L. Caplan, G.E. Linthorst, R.J. Desnick, and G. International Collaborative Fabry Disease Study, Safety and efficacy of recombinant human alpha-galactosidase A--replacement therapy in Fabry's disease. *N Engl J Med*, 345: (1) 9-16, 2001.

5. Schiffmann, R., J.B. Kopp, H.A. Austin, 3rd, S. Sabnis, D.F. Moore, T. Weibel, J.E. Balow, and R.O. Brady, Enzyme replacement therapy in Fabry disease: a randomized controlled trial. *JAMA*, 285: (21) 2743-9, 2001.

6. Abe, A., S. Gregory, L. Lee, P.D. Killen, R.O. Brady, A. Kulkarni, and J.A. Shayman, Reduction of globotriaosylceramide in Fabry disease mice by substrate deprivation. *J Clin Invest*, 105: (11) 1563-71, 2000.

7. Germain DP, Giugliani R, Hughes DA, Mehta A, Nicholls K, Barisoni L, Jennette CJ, Bragat A, Castelli J, Sitaraman S, Lockhart DJ, Boudes PF. Safety and pharmacodynamic effects of a pharmacological chaperone on α-galactosidase A activity and globotriaosylceramide clearance in Fabry disease: report from two phase 2 clinical studies. Orphanet J Rare Dis. 2012 Nov 24;7:91.

8. Moore, D.F., M. Ries, E.L. Forget, and R. Schiffmann, Enzyme replacement therapy in orphan and ultra-orphan diseases: the limitations of standard economic metrics as exemplified by Fabry-Anderson disease. *Pharmacoeconomics*, 25: (3) 201-8, 2007.

9. Desnick, R.J., R. Brady, J. Barranger, A.J. Collins, D.P. Germain, M. Goldman, G. Grabowski, S. Packman, and W.R. Wilcox, Fabry disease, an under-recognized multisystemic disorder: expert recommendations for diagnosis, management, and enzyme replacement therapy. *Ann Intern Med*, 138: (4) 338-46, 2003.

10. Germain, D.P., S. Waldek, M. Banikazemi, D.A. Bushinsky, J. Charrow, R.J. Desnick, P. Lee, T. Loew, A.C. Vedder, R. Abichandani, W.R. Wilcox, and N. Guffon, Sustained, long-term renal stabilization after 54 months of agalsidase beta therapy in

patients with Fabry disease. *J Am Soc Nephrol*, 18: (5) 1547-57, 2007.

11. Weidemann, F., M. Niemann, F. Breunig, S. Herrmann, M. Beer, S. Stork, W. Voelker, G. Ertl, C. Wanner, and J. Strotmann, Long-term effects of enzyme replacement therapy on fabry cardiomyopathy: evidence for a better outcome with early treatment. *Circulation*, 119: (4) 524-9, 2009.

12 .West, M., K. Nicholls, A. Mehta, J.T. Clarke, R. Steiner, M. Beck, B.A. Barshop, W. Rhead, R. Mensah, M. Ries, and R. Schiffmann, Agalsidase alfa and kidney dysfunction in Fabry disease. *J Am Soc Nephrol*, 20: (5) 1132-9, 2009.

13. Rosenberg, S.A., P. Aebersold, K. Cornetta, A. Kasid, R.A. Morgan, R. Moen, E.M. Karson, M.T. Lotze, J.C. Yang, S.L. Topalian, and et al., Gene transfer into humans--immunotherapy of patients with advanced melanoma, using tumor-infiltrating lymphocytes modified by retroviral gene transduction. *N Engl J Med*, 323: (9) 570-8, 1990.

14 .Ginn, S.L., I.E. Alexander, M.L. Edelstein, M.R. Abedi, and J. Wixon, Gene therapy clinical trials worldwide to 2012 - an update. *J Gene Med*, 15: (2) 65-77, 2013.

15. Levine, B.L., L.M. Humeau, J. Boyer, R.R. MacGregor, T. Rebello, X. Lu, G.K. Binder, V. Slepushkin, F. Lemiale, J.R. Mascola, F.D. Bushman, B. Dropulic, and C.H. June, Gene transfer in humans using a conditionally replicating lentiviral vector. *Proc Natl Acad Sci U S A*, 103: (46) 17372-7, 2006.

16. Manilla, P., T. Rebello, C. Afable, X. Lu, V. Slepushkin, L.M. Humeau, K. Schonely, Y. Ni, G.K. Binder, B.L. Levine, R.R. MacGregor, C.H. June, and B. Dropulic, Regulatory considerations for novel gene therapy products: a review of the process leading to the first clinical lentiviral vector. *Hum Gene Ther*, 16: (1) 17-25, 2005.

17. Cartier, N., S. Hacein-Bey-Abina, C.C. Bartholomae, G. Veres, M. Schmidt, I. Kutschera, M. Vidaud, U. Abel, L. Dal-Cortivo, L. Caccavelli, N. Mahlaoui, V. Kiermer, D. Mittelstaedt, C. Bellesme, N. Lahlou, F. Lefrere, S. Blanche, M. Audit, E. Payen, P. Leboulch, B. l'Homme, P. Bougneres, C. Von Kalle, A. Fischer, M. Cavazzana-Calvo, and P. Aubourg, Hematopoietic stem cell gene therapy with a lentiviral vector in X linked adrenoleukodystrophy. *Science*, 326: (5954) 818-23, 2009.

18. Biffi, A., E. Montini, L. Lorioli, M. Cesani, F. Fumagalli, T. Plati, C. Baldoli, S. Martino, A. Calabria, S. Canale, F. Benedicenti, G. Vallanti, L. Biasco, S. Leo, N. Kabbara, G. Zanetti, W.B. Rizzo, N.A. Mehta, M.P. Cicalese, M. Casiraghi, J.J. Boelens, U. Del Carro, D.J. Dow, M. Schmidt, A. Assanelli, V. Neduva, C. Di Serio, E. Stupka, J. Gardner, C. von Kalle, C. Bordignon, F. Ciceri, A. Rovelli, M.G. Roncarolo, A. Aiuti, M. Sessa, and L. Naldini, Lentiviral hematopoietic stem cell gene therapy benefits metachromatic leukodystrophy. *Science*, 341: (6148) 1233158, 2013.

19. Aiuti, A., L. Biasco, S. Scaramuzza, F. Ferrua, M.P. Cicalese, C. Baricordi, F.

Dionisio, A. Calabria, S. Giannelli, M.C. Castiello, M. Bosticardo, C. Evangelio, A. Assanelli, M. Casiraghi, S. Di Nunzio, L. Callegaro, C. Benati, P. Rizzardi, D. Pellin, C. Di Serio, M. Schmidt, C. Von Kalle, J. Gardner, N. Mehta, V. Neduva, D.J. Dow, A. Galy, R. Miniero, A. Finocchi, A. Metin, P.P. Banerjee, J.S. Orange, S. Galimberti, M.G. Valsecchi, A. Biffi, E. Montini, A. Villa, F. Ciceri, M.G. Roncarolo, and L. Naldini, Lentiviral hematopoietic stem cell gene therapy in patients with Wiskott-Aldrich syndrome. *Science*, 341: (6148) 1233151, 2013.

20. Medin, J.A., M. Tudor, R. Simovitch, J.M. Quirk, S. Jacobson, G.J. Murray, and R.O. Brady, Correction in trans for Fabry disease: expression, secretion and uptake of alpha-galactosidase A in patient-derived cells driven by a high-titer recombinant retroviral vector. *Proc Natl Acad Sci U S A*, 93: (15) 7917-22, 1996.

21. Yoshimitsu, M., T. Sato, K. Tao, J.S. Walia, V.I. Rasaiah, G.T. Sleep, G.J. Murray, A.G. Poeppl, J. Underwood, L. West, R.O. Brady, and J.A. Medin, Bioluminescent imaging of a marking transgene and correction of Fabry mice by neonatal injection of recombinant lentiviral vectors. *Proc Natl Acad Sci U S A*, 101: (48) 16909-14, 2004.

22. Higuchi, K., M. Yoshimitsu, X. Fan, X. Guo, V.I. Rasaiah, J. Yen, C. Tei, T. Takenaka, and J.A. Medin, Alpha-galactosidase A-Tat fusion enhances storage reduction in hearts and kidneys of Fabry mice. *Mol Med*, 16: (5-6) 216-21, 2010.

23. Liang, S.B., M. Yoshimitsu, A. Poeppl, V.I. Rasaiah, J. Cai, D.H. Fowler, and J.A. Medin, Multiple reduced-intensity conditioning regimens facilitate correction of Fabry mice after transplantation of transduced cells. *Mol Ther*, 15: (3) 618-27, 2007.

24. Pacienza, N., M. Yoshimitsu, N. Mizue, B.C. Au, J.C. Wang, X. Fan, T. Takenaka, and J.A. Medin, Lentivector transduction improves outcomes over transplantation of human HSCs alone in NOD/SCID/Fabry mice. *Mol Ther*, 20: (7) 1454-61, 2012.

25. Qin, G., T. Takenaka, K. Telsch, L. Kelley, T. Howard, T. Levade, R. Deans, B.H. Howard, H.L. Malech, R.O. Brady, and J.A. Medin, Preselective gene therapy for Fabry disease. *Proc Natl Acad Sci U S A*, 98: (6) 3428-33, 2001.

26. Scaife, M., N. Pacienza, B.C. Au, J.C. Wang, S. Devine, E. Scheid, C.J. Lee, O. Lopez-Perez, A. Neschadim, D.H. Fowler, R. Foley, and J.A. Medin, Engineered human Tmpk fused with truncated cell-surface markers: versatile cell-fate control safety cassettes. *Gene Ther*, 20: (1) 24-34, 2013.

27. Takenaka, T., G.J. Murray, G. Qin, J.M. Quirk, T. Ohshima, P. Qasba, K. Clark, A.B. Kulkarni, R.O. Brady, and J.A. Medin, Long-term enzyme correction and lipid reduction in multiple organs of primary and secondary transplanted Fabry mice receiving transduced bone marrow cells. *Proc Natl Acad Sci U S A*, 97: (13) 7515-20, 2000.

28. Takenaka, T., G. Qin, R.O. Brady, and J.A. Medin, Circulating alpha-galactosidase

A derived from transduced bone marrow cells: relevance for corrective gene transfer for Fabry disease. *Hum Gene Ther*, 10: (12) 1931-9, 1999.

29. Yoshimitsu, M., K. Higuchi, S. Ramsubir, T. Nonaka, V.I. Rasaiah, C. Siatskas, S.B. Liang, G.J. Murray, R.O. Brady, and J.A. Medin, Efficient correction of Fabry mice and patient cells mediated by lentiviral transduction of hematopoietic stem/progenitor cells. *Gene Ther*, 14: (3) 256-65, 2007.

30. Walia, J.S., A. Neschadim, O. Lopez-Perez, A. Alayoubi, X. Fan, S. Carpentier, M. Madden, C.J. Lee, F. Cheung, D.A. Jaffray, T. Levade, J.A. McCart, and J.A. Medin, Autologous transplantation of lentivector/acid ceramidase-transduced hematopoietic cells in nonhuman primates. *Hum Gene Ther*, 22: (6) 679-87, 2011.

31. Banikazemi, M., J. Bultas, S. Waldek, W.R. Wilcox, C.B. Whitley, M. McDonald, R. Finkel, S. Packman, D.G. Bichet, D.G. Warnock, R.J. Desnick, and G. Fabry Disease Clinical Trial Study, Agalsidase-beta therapy for advanced Fabry disease: a randomized trial. *Ann Intern Med*, 146: (2) 77-86, 2007.

32. Mehta, A., M. Beck, P. Elliott, R. Giugliani, A. Linhart, G. Sunder-Plassmann, R. Schiffmann, F. Barbey, M. Ries, J.T. Clarke, and i. Fabry Outcome Survey, Enzyme replacement therapy with agalsidase alfa in patients with Fabry's disease: an analysis of registry data. *Lancet*, 374: (9706) 1986-96, 2009.

33. Rombach, S.M., B.E. Smid, M.G. Bouwman, G.E. Linthorst, M.G. Dijkgraaf, and C.E. Hollak, Long term enzyme replacement therapy for Fabry disease: effectiveness on kidney, heart and brain. *Orphanet J Rare Dis*, 8: 47, 2013.

34.Auray-Blais, C., D. Cyr, A. Ntwari, M.L. West, J. Cox-Brinkman, D.G. Bichet, D.P. Germain, R. Laframboise, S.B. Melancon, T. Stockley, J.T. Clarke, and R. Drouin, Urinary globotriaosylceramide excretion correlates with the genotype in children and adults with Fabry disease. *Mol Genet Metab*, 93: (3) 331-40, 2008.

35. Naldini, L., U. Blomer, P. Gallay, D. Ory, R. Mulligan, F.H. Gage, I.M. Verma, and D. Trono, In vivo gene delivery and stable transduction of nondividing cells by a lentiviral vector. *Science*, 272: (5259) 263-7, 1996.

36. Auray-Blais, C. and M. Boutin, Novel Gb(3) isoforms detected in urine of Fabry disease patients: a metabolomic study. *Curr Med Chem*, 19: (19) 3241-52, 2012.

37. Auray-Blais, C., A. Ntwari, J.T. Clarke, D.G. Warnock, J.P. Oliveira, S.P. Young, D.S. Millington, D.G. Bichet, S. Sirrs, M.L. West, R. Casey, W.L. Hwu, J.M. Keutzer, X.K. Zhang, and R. Gagnon, How well does urinary lyso-Gb3 function as a biomarker in Fabry disease? *Clin Chim Acta*, 411: (23-24) 1906-14, 2010.

38. Boutin, M., R. Gagnon, P. Lavoie, and C. Auray-Blais, LC-MS/MS analysis of plasma lyso-Gb3 in Fabry disease. *Clin Chim Acta*, 414: 273-80, 2012.

39. Qasim, W., C.A. Vink, and A.J. Thrasher, Hybrid lentiviral vectors. *Mol Ther*, 18: (7) 1263-7, 2010.

40. Cockrell, A.S. and T. Kafri, Gene delivery by lentivirus vectors. *Mol Biotechnol*, 36: (3) 184-204, 2007.

41. Follenzi, A., L. Santambrogio, and A. Annoni, Immune responses to lentiviral vectors. *Curr Gene Ther*, **7**: (5) 306-15, 2007.

42 .CHMP public assessment report on the shortage of Fabrazyme Nov. 19, 2010. http://www.ema.europa.eu/docs/en_GB/document_library/Other/2010/11/WC500099241.pdf

43. Crump, M. and A. Keating, *Autologous bone marrow transplantation: high dose etoposide, melphalan, total body irradiation and ABMT in patients with hematologic maliganancies (Update)*, in *Critical Papers in Bone Marrow Transplantation 1992: An Anthology*, R. Powles and E.C. Gordon-Smith, Editors. 1992. p. 294-298.

44. Mollee, P.N., A.D. Wechalekar, D.L. Pereira, N. Franke, D. Reece, C. Chen, and A.K. Stewart, Autologous stem cell transplantation in primary systemic amyloidosis: the impact of selection criteria on outcome. *Bone Marrow Transplant*, 33: (3) 271-7, 2004.

45. Bodensteiner, D., C.R. Scott, K.B. Sims, G.M. Shepherd, R.D. Cintron, and D.P. Germain, Successful reinstitution of agalsidase beta therapy in Fabry disease patients with previous IgE-antibody or skin-test reactivity to the recombinant enzyme. *Genet Med*, 10: (5) 353-8, 2008.

46. Deegan, P.B., Fabry disease, enzyme replacement therapy and the significance of antibody responses. *J Inherit Metab Dis*, 35: (2) 227-43, 2012.

47. Linthorst, G.E., C.E. Hollak, W.E. Donker-Koopman, A. Strijland, and J.M. Aerts, Enzyme therapy for Fabry disease: neutralizing antibodies toward agalsidase alpha and beta. *Kidney Int*, 66: (4) 1589-95, 2004.

48. Rombach, S.M., J.M. Aerts, B.J. Poorthuis, J.E. Groener, W. Donker-Koopman, E. Hendriks, M. Mirzaian, S. Kuiper, F.A. Wijburg, C.E. Hollak, and G.E. Linthorst, Long term effect of antibodies against infused alpha-galactosidase A in Fabry disease on plasma and urinary (lyso)Gb3 reduction and treatment outcome. *PLoS One*, 7: (10) e47805, 2012.

49. Wilcox, W.R., G.E. Linthorst, D.P. Germain, U. Feldt-Rasmussen, S. Waldek, S.M. Richards, D. Beitner-Johnson, M. Cizmarik, J.A. Cole, W. Kingma, and D.G. Warnock, Anti-alpha-galactosidase A antibody response to agalsidase beta treatment: data from the Fabry Registry. *Mol Genet Metab*, 105: (3) 443-9, 2012.

50. Lenders, M., J. Stypmann, T. Duning, B. Schmitz, S.M. Brand, and E. Brand, Serum Mediated Inhibition of Enzyme Replacement Therapy in Fabry Disease. *J Am Soc Nephrol*, 2015.

51. Lenders M et al. Serum-mediated inhibition of enzyme replacement therapy in Fabry disease. J Am Soc Nephrol 2016;27: 256–264

52. Deegan PB. Fabry disease, enzyme replacement therapy and the significance of antibody responses. J Inherit Metab Dis 2011; doi 10.1007/s10545-011-9400-y

53. Smid BE et al. Consequences of a global enzyme shortage of agalsidase beta in adult Dutch Fabry patients. Orphanet Journal of Rare Diseases 2011;6:69. doi:10.1186/1750-1172-6-69

54. Ghali J et al. Effect of Reduced Agalsidase Beta Dosage in Fabry Patients: The Australian Experience. J Inherit Metab Dis Reports. 2011; doi 10.1007/8904_2011_44

55. Personal communication Dr. Michael L. West Department of Medicine, Dalhousie University, Halifax NS, Canada.

56. Arends M et al. Retrospective study of long-term outcomes of enzyme replacement therapy in Fabry disease: Analysis of prognostic factors PLoS ONE 2017;12(8):e0182379. doi.org/10.1371/journal.pone.0182379

57. Auray-Blais C., Blais C.M., Ramaswami U., Boutin M., Germain D.P., Dyack S., Bodamer O., Pintos-Morell G., Clarke J.T., Bichet D.G., Echevarria L., West M.L., Lavoie P, Urinary biomarker investigation in children with Fabry disease using tandem mass spectrometry. *Clin Chim Acta*, 438:195-204, 2015

58. Boutin M., Lavoie P., Abaoui M., Auray-Blais C., Tandem mass spectrometry quantitation of lyso-Gb_3 and six related analogs in plasma for Fabry disease patients, *Curr Protoc Hum Genet*, 90:17.23.1-9, 2016.

59. Zufferey, R., D. Nagy, R.J. Mandel, L. Naldini, and D. Trono, Multiply attenuated lentiviral vector achieves efficient gene delivery in vivo. *Nat Biotechnol*, 15: (9) 871-5, 1997.

60. Cornetta, K., J. Yao, A. Jasti, S. Koop, M. Douglas, D. Hsu, L.A. Couture, T. Hawkins, and L. Duffy, Replication-competent lentivirus analysis of clinical grade vector products. *Mol Ther*, 19: (3) 557-66, 2011.

61. Boutin M, Auray-Blais C. Metabolomic Discovery of Novel Urinary Galabiosylceramide Analogs as Fabry Disease Biomarkers. *J Am Mass Spectrom*. 2015 26:499-510.

62. Oken, M.M., R.H. Creech, D.C. Tormey, J. Horton, T.E. Davis, E.T. McFadden, and P.P. Carbone, Toxicity and response criteria of the Eastern Cooperative Oncology

Group. *Am J Clin Oncol*, 5: (6) 649-55, 1982.

57. Abaoui M., Boutin M., Lavoie P., Auray-Blais C, Tandem mass spectrometry multiplex analysis of methylated and non-methylated urinary Gb_3 isoforms in Fabry disease patients. *Clin Chim Acta*, 452:191-8, 2016.

58.Lavoie P., Boutin M., Abaoui M., Auray-Blais C., Fabry disease biomarkers : Analysis of urinary lyso-Gb_3 and seven related analogs using tandem mass spectrometry, Curr Protoc Hum Genet, 90 :17.22.1-17.22.12, 2016.

59. Boutin M., Auray-Blais C., Multiplex tandem mass spectrometry analysis of novel plasma lyso-Gb_3-related analogues in Fabry disease, *Anal Chem*, 86(7):3476-83, 2014.
